# Targeted Metabolite Fingerprints of Thirteen *Gambierdiscus*, Five *Coolia* and Two *Fukuyoa* Species

**DOI:** 10.3390/md22030119

**Published:** 2024-03-02

**Authors:** J. Sam Murray, Emillie M. F. Passfield, Lesley L. Rhodes, Jonathan Puddick, Sarah C. Finch, Kirsty F. Smith, Roel van Ginkel, Elizabeth M. Mudge, Tomohiro Nishimura, Hiroshi Funaki, Masao Adachi, Michèle R. Prinsep, D. Tim Harwood

**Affiliations:** 1 Cawthron Institute, Private Bag 2, Nelson 7040, New Zealand; emillie.passfield@cawthron.org.nz (E.M.F.P.); lesley.rhodes@cawthron.org.nz (L.L.R.); jonathan.puddick@cawthron.org.nz (J.P.); kirsty.smith@cawthron.org.nz (K.F.S.); roel.vanginkel@cawthron.org.nz (R.v.G.); nishimura_tomohiro49@fra.go.jp (T.N.); tim.harwood@cawthron.org.nz (D.T.H.); 2 New Zealand Food Safety Science and Research Centre, Massey University, Private Bag 11 222, Palmerston North 4442, New Zealand; 3 School of Science, University of Waikato, Private Bag 3105, Hamilton 3240, New Zealand; michele.prinsep@waikato.ac.nz; 4 AgResearch, Ruakura Research Centre, Private Bag 3123, Hamilton 3240, New Zealand; sarah.finch@agresearch.co.nz; 5 Biotoxin Metrology, National Research Council Canada, 1411 Oxford Street, Halifax, NS B3H 3Z1, Canada; elizabeth.mudge@nrc-cnrc.gc.ca; 6 Japan Fisheries Research and Education Agency, 2-17-5 Maruishi, Hatsukaichi 739-0452, Hiroshima, Japan; 7 Faculty of Agriculture and Marine Science, Kochi University, 200 Otsu, Monobe, Nankoku 783-8502, Kochi, Japan; h-funaki@kochi-u.ac.jp (H.F.); madachi@kochi-u.ac.jp (M.A.)

**Keywords:** ciguatera poisoning, ciguatoxin, maitotoxin, bioactive, mass spectrometry, benthic dinoflagellate, toxicity

## Abstract

The genus *Gambierdiscus* produces an array of bioactive hydrophilic and lipophilic secondary metabolites that range in mode of action and toxicity. In this study, the metabolite fingerprint was mapped for thirteen *Gambierdiscus*, five *Coolia* and two *Fukuyoa* species (34 isolates) by assessing the production of 56 characterised secondary metabolites. *Gambierdiscus polynesiensis* was the only species to produce Pacific-ciguatoxin-3B (P-CTX3B), P-CTX3C, iso-P-CTX3B/C, P-CTX4A, P-CTX4B and iso-P-CTX4A/B. *G. australes* produced maitotoxin-1 (MTX-1) and MTX-5, *G. cheloniae* produced MTX-6 and *G. honu* produced MTX-7. Ubiquitous production of 44-methylgambierone was observed amongst all the *Gambierdiscus* isolates, with nine species also producing gambierone. Additional gambierone analogues, including anhydrogambierone (tentatively described herein), were also detected in all *Gambierdiscus* species, two *Coolia* and two *Fukuyoa* species. Gambieroxide was detected in *G. lewisii* and *G. pacificus* and gambieric acid A was detected in ten *Gambierdiscus* species, with *G. australes* (CAWD381) being the only isolate to produce gambieric acids A–D. This study has demonstrated that the isolates tested to date produce the known CTXs or MTXs, but not both, and highlighted several species that produced ‘unknown’ compounds displaying characteristics of cyclic polyethers, which will be the focus of future compound discovery efforts.

## 1. Introduction

Ciguatera poisoning (CP) is prevalent in all circumtropical regions of the world [1,2] and poses a major seafood safety risk to coastal communities, particularly those in the tropical and sub-tropical latitudes of the South Pacific Basin [3,4]. While the existence of CP has been known for centuries [5,6,7], with the first historical intoxication event being reported in 1521 [8], the true number of intoxications and impact from CP are not known. It is estimated that 10,000–50,000 people are affected annually, but epidemiological studies indicate that this number is underestimated and represents <20% of actual cases [7].

*Gambierdiscus* is a benthic dinoflagellate that grows on a variety of macroalgal species, seagrasses, coral substrate and rubble, and volcanic sands [9]. *Gambierdiscus* is regarded as an opportunistic dinoflagellate that proliferates following damage to the reef system from tropical hurricanes, outbreaks of crown of thorn starfish (*Acanthaster planci*), coral bleaching events or anthropogenic activities (e.g., nutrient runoff that encourages macroalgal growth) [10,11,12,13].

Until 1999, *Gambierdiscus* was regarded as a monotypic genus, with all *Gambierdiscus* cells recorded as *G. toxicus*. However, in recent years there has been an increased international focus on CP and, coupled with extensive use of molecular methods for taxonomic assessments, the number of new species has grown rapidly. To date, 19 species of *Gambierdiscus* have been described, along with several undescribed ribotypes, making it one of the largest genera of marine benthic dinoflagellates. Species include *G. australes*, *G. balechii*, *G. belizeanus*, *G. caribaeus*, *G. carolinianus*, *G. carpenteri*, *G. cheloniae*, *G. excentricus*, *G. holmesii*, *G. honu*, *G. jejuensis*, *G. lapillus*, *G. lewisii*, *G. pacificus*, *G. polynesiensis*, *G. scabrosus*, *G. silvae*, *G. toxicus* and *G. vietnamensis*. However, this list will undoubtedly continue to increase as seven of the species listed above were described within the past six years [14,15,16,17,18,19].

Adding to the complexity of CP is the fact that *Gambierdiscus* is commonly found in assemblages with benthic dinoflagellates from the genera *Fukuyoa* and *Coolia*. *Fukuyoa* has a close phylogenetic relationship with *Gambierdiscus* and was originally included in this genus. It was reclassified as *Fukuyoa* in 2015 [20] and includes four species: *F. koreensis*, *F. paulensis*, *F. ruetzleri* and *F. yasumotoi* [20,21,22]. The *Coolia* genus is genetically very distinct from *Gambierdiscus*, yet it is known to produce similar secondary metabolites [23]. Eight *Coolia* species have been described: *C. monotis*, *C. tropicalis*, *C. areolata*, *C. canariensis*, *C. malayensis*, *C. palmyrensis*, *C. santacroce* and *C. guanchica* [24]. It is, however, unclear if, and to what extent, the genera *Fukuyoa* and *Coolia* contribute to CP events.

**Figure 1:**
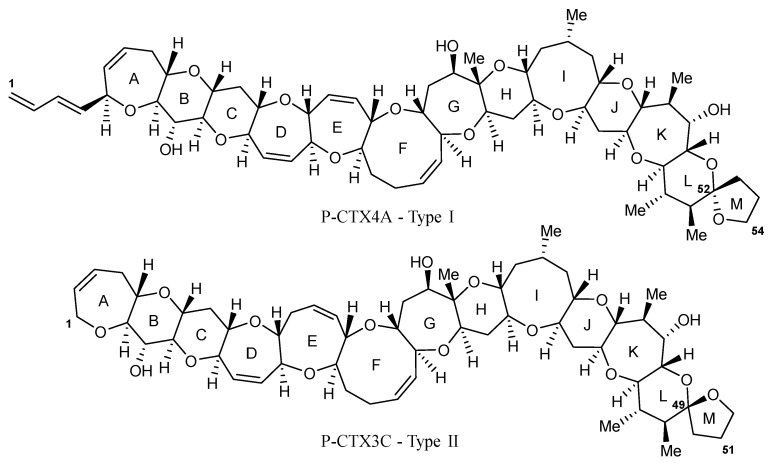
Example structures of P-CTX Type I (P-CTX4A), showing the aliphatic hydrocarbon chain on ring A, and Type II (P-CTX3C) with the eight membered ring E.

The genus *Gambierdiscus* produces a complex array of bioactive, cyclic polyether, hydrophilic and lipophilic metabolites that range in toxicity and mode of action. These include ciguatoxins (CTXs), maitotoxins (MTXs) and gambierones. CTXs are a class of lipophilic marine biotoxins that accumulate in fish species, marine invertebrates, such as echinoderms (e.g., urchin, *Tripneustes gratilla*, and starfish, *Ophidiaster ophidianus*), gastropods (e.g., cone snails, *Conus* spp.) and bivalve molluscs (e.g., the giant clam, *Tridacna maxima*) [25,26,27,28,29], along with octopus (*Octopus cyanea*) and crustaceans (e.g., crab, *Percnon* spp., and lobster, *Panulirus penicillatus*) [30,31]. These metabolites are odourless, tasteless, heat stable, resistant to gastric degradation [1] and represent a structurally diverse toxin class, which have historically been described based on geographical origin [12]. This includes the Pacific region (P-CTXs), where more than 20 P-CTX analogues have been identified. Within the P-CTX class, there are two distinct structural types: Type I (e.g., P-CTX4A; Figure 1) and Type II (e.g., P-CTX3C; Figure 1) [32,33]. Both contain a continuous 13 ether ring (A–M) fused backbone, with the differences being the aliphatic hydrocarbon chain on ring A for Type I and the size of ring E (seven-membered for Type I or eight-membered for Type II). CTXs bind to site five of the voltage-gated sodium channel (VGSC) [34], causing an influx of sodium ions into the cell, resulting in a depolarization of the cell membrane [35]. It is acknowledged that the naming convention of CTXs is likely to change in the future and will exclude geographical nomenclature for the Pacific [36]. This is due to the undoubted overlap of P-CTXs appearing in fish from outside the Pacific region.

**Figure 2:**
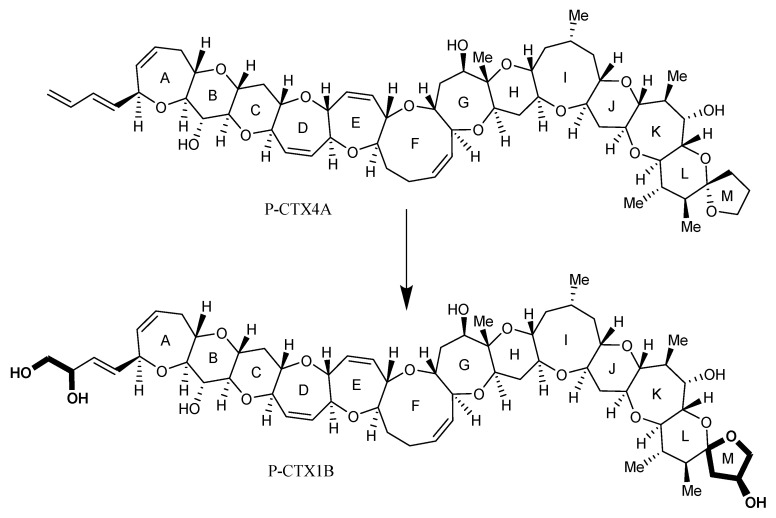
Scheme depicting the spiroisomerisation and oxidation modifications of P-CTX4A to form P-CTX1B (in bold).

During biomagnification of the algal P-CTX metabolites in the marine food web, biotransformation occurs, creating more toxic analogues. For example, it has been demonstrated in vitro that the Type I algal epimers P-CTX4A/B undergo acid-catalysed spiroisomerisation and oxidative modifications to form, for example, 52-*epi*-54-deoxy-CTX1B, 54-deoxy-CTX1B and P-CTX1B (Figure 2) [33], whereas the Type II algal metabolite P-CTX3C undergoes oxidative modifications to form, for example, 2-hydroxy-CTX3C, 2,3-dihydroxy-CTX3C and 51-hydroxy-CTX3C [33]. Toxicological assessment using purified material showed that P-CTX1B, 52-*epi*-54-deoxy-CTX1B and 51-hydroxy-CTX3C were four to ten times more toxic than their algal precursors (P-CTX4A/B and P-CTX3C, respectively) when tested on mice via intraperitoneal (i.p.) injection, using the neuroblastoma-cell-based assay (N2a-CBA) and the radiolabelled receptor binding assay (RBA) [32]. Of all the analogues tested, P-CTX1B was the most toxic when assessed using N2a-CBA and 51-hydroxy-CTX3C was the most toxic via i.p. injection. Caribbean ciguatoxins (C-CTXs) currently comprise of five analogues: two epimers at C-56 (C-CTX1/2), each with a continuous 14 ether ring trans-fused backbone [37], the respective N-seco forms (C-CTX3/4) [38], and C-CTX5, which has a C-3 ketone rather than an hydroxyl group [39] and is an algal precursor that is produced by *G. silvae* and *G. caribaeus*. Other CTXs have been detected in fish caught in the Indian Ocean (I-CTX1-6) [40], although the chemical structures of these I-CTX analogues have not been characterised and their toxicity is unknown.

**Figure 3:**
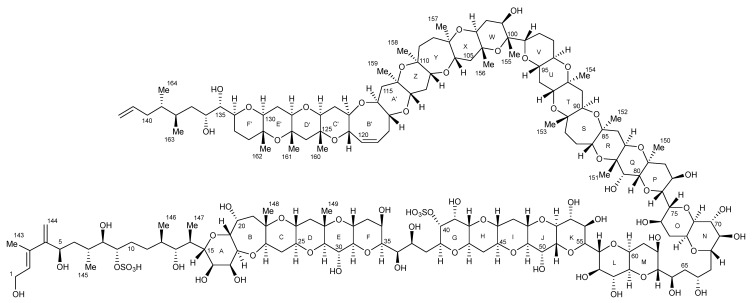
Structure of maitotoxin-1 (adapted from Murata et al., 1993) [41].

MTXs represent a class of hydrophilic, mono- or di-sulphated metabolites comprised of 32 fused rings and an aliphatic hydrocarbon chain at each terminus. MTX-1 (Figure 3) is both the largest natural non-biopolymer and most toxic non-peptide compound known (LD_50_ value of 50 ng/kg to mice via i.p. injection), although oral potency is considerably lower than by i.p. injection [41]. This metabolite activates the extracellular calcium channels found in the cell membrane and causes an increase in cytosolic-free calcium ions [42], which leads to oncotic cell death through increased osmotic pressure exerted by protein complexes [43,44,45,46]. Since MTX-1 was identified in 1976 [47], six additional MTX analogues have been described from *Gambierdiscus* species. MTX-2 and MTX-3 were reported in 1994 as monosulphated variants [48]. MTX-3 was isolated based on published spectra in 2019 and structurally elucidated as 44-methylgambierone (44-MG) [49], with this finding confirmed by a second research group two months later [50]. MTX-4 was identified in isolates of *G*. *excentricus* from the Canary Islands [51], and MTX-5 was identified in *G. australes* isolates from the Mediterranean Sea [52]. MTX-6 and MTX-7 are monosulphated analogues similar to MTX-1 and are produced by isolates of *G. cheloniae* and *G. honu*, respectively [53].

**Figure 4:**
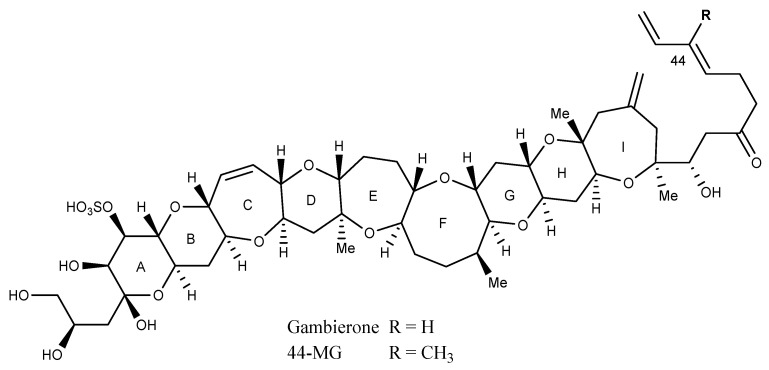
Structures of gambierone and 44-methylgambierone (previously reported as MTX-3; adapted from Murray et al., 2019) [49].

The hydrophilic gambierones [49,54,55,56] consist of nine continuous trans-fused ether rings and are either mono- or di-sulphated. Some analogues have a terminal diol, 1,3 diene, saturated double bonds and/or are deoxygenated (Figure 4) [56]. The production of 44-MG (originally referred to as MTX-3) is ubiquitous to all *Gambierdiscus* species tested to date [57], while only some of these species produce gambierone. Some *Fukuyoa* and *Coolia* species also produce 44-MG and gambierone. It has been reported that gambierone displays the same VGSC antagonism as P-CTX-3C, yet when 44-MG and gambierone were assessed using an in vivo mouse model [23,57] and N2a-CBA [58], they showed significantly lower toxicity compared to the P-CTXs.

**Figure 5:**
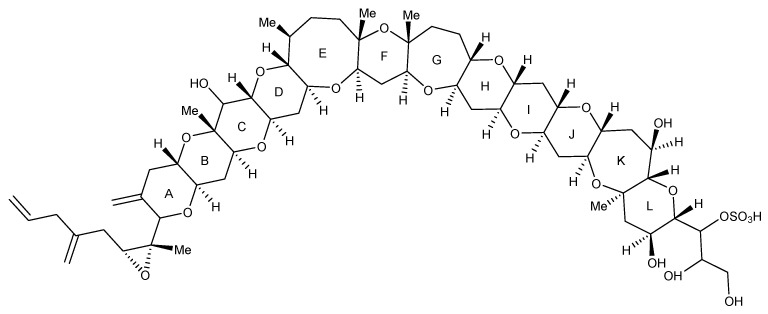
Structure of gambieroxide (adapted from Watanabe et al., 2013) [59].

Other metabolites produced by *Gambierdiscus* include gambieroxide (Figure 5) [59], a metabolite with 12 continuous trans-fused ether rings, a sulphate ester, an epoxide and an olefinic sidechain [59]. Currently, there is no toxicity information available on gambieroxide.

**Figure 6:**
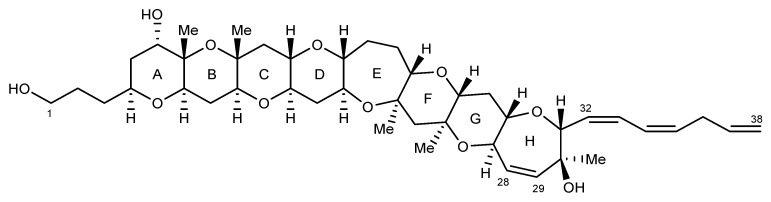
Structure of gambierol (adapted from Morohashi et al., 1999) [60].

Gambierol (Figure 6) [60] is a very lipophilic analogue with eight continuous trans-fused ether rings and a heptatriene sidechain. It displays potent neurotoxicity against mice when administered by i.p. injection, with neurological symptoms similar to those exhibited in CP events. It is a potent voltage-gated potassium channel blocker [61,62], as well as a functional antagonist of site five in neuronal VGSCs. The latter has a similar mode of action as the P-CTXs. Two independent studies reported that the C-28–C-29 double bond in the seven-membered ring H, and the unsaturated hydrocarbon sidechain C-32–C-38, are structural characteristics crucial for the potent toxicity of gambierol [63,64].

**Figure 7:**
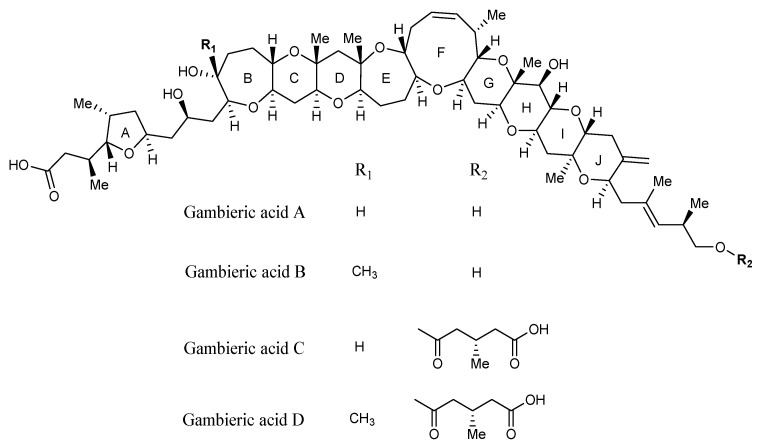
Structures of the four described gambieric acid analogues (adapted from Morohashi et al., 2000) [65].

Lastly, gambieric acids consist of nine continuous trans-fused rings. Four analogues have been characterised: gambieric acid A and B and the 3-methylhemiglutarate forms, gambieric acid C and D, each containing a carboxylic acid functionality (Figure 7) [65,66]. These compounds displayed strong antifungal activity, yet had no toxicity to mice via i.p. injection [67]. It is unknown if other secondary metabolites produced by *Gambierdiscus* display the same antifungal activity.

In 2018, the World Health Organization and the Food and Agriculture Organization of the United Nations hosted an expert meeting to synthesize the multi-disciplinary knowledge on CP. The primary outcome was a report that highlighted several priority research areas, with one being an improved understanding of the toxic secondary metabolites produced by *Gambierdiscus* species [36]. It is well documented that consumption of marine species contaminated with P-CTXs will lead to CP [25,26,27,29,68], while the contribution of the other secondary metabolites remains unclear. Adding to the complexity of CP, only *G. polynesiensis* has been definitively shown to produce P-CTXs using liquid chromatography–tandem mass spectrometry (LC–MS/MS), yet the known (limited) distribution of this species does not align with global intoxication events. In addition, most species display some bioactivity when using in vitro and in vivo assay techniques [69]. It is therefore crucial that both the known and unknown compounds are identified to understand their role in CP and whether they could pose a risk to human health.

In this study, we used targeted LC–MS/MS analysis to map the characterised metabolite fingerprint of 34 benthic dinoflagellate isolates representing thirteen *Gambierdiscus*, five *Coolia* and two *Fukuyoa* species. The isolates originated from Aotearoa/New Zealand, Australia, Hong Kong, Japan, Pohnpei, Rangitāhua Kermadec Islands, Rarotonga, Spain, St. Barthelemy Island and Tonga, and are either contained in the Cawthron Institute Culture Collection of Microalgae (New Zealand), at Kochi University (Japan) or historical microalgal extracts stored at Cawthron Institute. Each species was analysed for 56 hydrophilic and lipophilic bioactive metabolites that have been reported in the literature, including three tentative gambierone analogues that are described herein for the first time. For the majority of these metabolites, reference material was not available, making detections of said metabolites tentative. During the analysis, an array of ‘unknown’ cyclic polyethers were also identified based on characteristic mass spectral patterns, and when coupled with known toxicity information of the isolates, several species of interest have been highlighted and will become the focus of future compound discovery efforts.

## 2. Results

### 2.1. Ciguatoxins

The isolates were quantitatively analysed for six algal P-CTX metabolites, including two isomers, and qualitatively assessed for the production of three M-seco variants (M-seco-CTX3B/C, M-seco-CTX3B/C acetate and M-seco-CTX4A/B). Only the *G. polynesiensis* isolates CAWD212 and CAWD267 produced the algal P-CTX metabolites (LoD = 0.01 pg/cell), both displaying similar profiles for P-CTX3B, P-CTX3C, P-CTX4A and P-CTX4B (Table 1 and Table S1). The cell quotas of these four analogues were quantified using LC–MS/MS calibrated reference material and ranged from 0.02 to 1.1 pg/cell. The most abundant P-CTX analogue was P-CTX3B, with the highest cell quota (1.1 pg/cell) observed from *G. polynesiensis* CAWD212. The two *G. polynesiensis* isolates also produced earlier eluting isomers of both P-CTX3B/C and P-CTX4A/B. These compounds were more abundant than the primary algal CTX metabolites and were quantified using P-CTX3B and P-CTX4A calibration standards, respectively, and with an assumed relative response factor of 1. Cell quotas for the isomers ranged from 1.3 to 7.8 pg/cell, with the highest observed for iso-P-CTX3B/C in *G. polynesiensis* CAWD267 (7.8 pg/cell).

There were no detections of the 18 fish CTX metabolites investigated in the algal isolates that aligned with the literature and/or reference material. These included P-CTX1B, 52-*epi*-54-deoxy-CTX1B, 54-deoxy-CTX1B, 51-hydroxy-CTX3C, 2,3-dihydroxy-CTX3B, 2,3-dihydroxy-CTX3C, 2-hydroxy-CTX3C, 51-hydroxy-2-oxo-CTX3C, A-seco-51-hydroxy-CTX3C, 2,3,51-trihydroxy-CTX3C, 7-oxo-CTX1B, 7-hydroxy-CTX1B, 4-hydroxy-7-oxo-CTX1B, CTX1A, 52-*epi*-CTX1B, 54-*epi*-CTX1B, 54-*epi*-52-*epi*-CTX1B and 54-deoxy-50-hydroxy-CTX1B.

The algal isolates were also analysed for C-CTX1/2, C-CTX3/4, C-CTX5, I-CTX1/2, I-CTX3/4, I-CTX5 and I-CTX6 (Table S2), with no detections that aligned with the literature.

### 2.2. Maitotoxins

The production of MTX-1, MTX-2, MTX-4, MTX-5, MTX-6 and MTX-7 was investigated, with cell quotas calculated for the six analogues (Table 1 and Table S3). *G. australes* CAWD149 and CAWD381 were the only isolates to produce MTX-1 (6 and 9 pg/cell, respectively) and MTX-5 (0.2 and 0.1 pg/cell, respectively), *G. cheloniae* CAWD232 and CAWD236 produced MTX-6 (4 and 5 pg/cell, respectively) and *G. honu* CAWD242 and CAWD250 produced MTX-7 (14 and 2 pg/cell, respectively). No MTX-2 or MTX-4 were detected in the isolates; however, as no reference material (purified standard or a positive culture extract) was available for this study, this result cannot be confirmed.

A compound related to MTX-6 was observed in the *G. pacificus* CAWD227 and CAWD337 isolates. However, it was later eluting than MTX-6 (2.83 min vs. 2.59 min) and had a different multiple reaction monitoring (MRM) confirmation ratio (4.5 vs. 2.9). In addition, a peak was observed in the *G. carpenteri* CAWD237 trace, although the confirmation ratio was higher (6.1 vs. 2.9), and this isolate has been demonstrated to be of low toxicity by i.p. injection in mice [69]. A compound related to MTX-7 was observed in the *G. caribaeus* CAWD301 trace. It had the same confirmation ratio but was later eluting than that of MTX-7 (2.62 min vs. 2.47 min).

### 2.3. Gambierones

Cell quotas for gambierone and 44-MG were calculated using an in-house purified qNMR reference standard (Table 1 and Table S4). 44-MG was observed in all 21 isolates of the thirteen *Gambierdiscus* species analysed, with the cell quotas for twelve isolates, representing ten species, reported previously in Murray et al. [23]. Cell quotas for 44-MG ranged from 5–441 pg/cell. *G. carpenteri* CAWD364 had the highest cell quota (441 pg/cell), followed by *G. lapillus* CAWD338 (270 pg/cell) and *G. australes* CAWD149 (259 pg/cell). Nine of the thirteen *Gambierdiscus* species also produced detectable levels of gambierone (LoD = 0.01 pg/cell). Cell quotas varied considerably, ranging from 1–540 pg/cell (Table S4), with the highest cell quota observed in *G. belizeanus* CCMP401 (540 pg/cell). The second highest producer was *G. cheloniae* CAWD236 (358 pg/cell), then *G. scabrosus* K070922_1 (166 pg/cell). The *G. jejuensis* NIES-4120 and *G. scabrosus* CAWD429 isolates were grown in two different media, with the cell quotas for 44-MG being very similar, with more gambierone produced by *G. scabrosus* CAWD429 when grown in f/8 as opposed to IMK/2 media. As published by Murray et al. [23], *C. malayensis*, *C. tropicalis*, *F. paulensis* and *F. ruetzleri* produce 44-MG (5–65 pg/cell), with only *C. malayensis* and *F. paulensis* producing gambierone (2–17 pg/cell). Gambierones were not detected in the *C. canariensis*, *C. monotis* or *C. palmyrensis* isolates.

The isolates were qualitatively assessed for the production of nine additional gambierone analogues, including anhydrogambierone, dianhydrogambierone and dianhydro-44-MG (Table 2 and Table S4). Anhydrogambierone was detected in eight *Gambierdiscus* species (*G. belizeanus*, *G. carpenteri*, *G. cheloniae*, *G. holmesii*, *G. honu*, *G. pacificus*, *G. polynesiensis* and *G. scabrosus*), *C. malayensis* CAWD154 and CAWD175 and *F. ruetzleri* S044 and S051. 38-Deoxy-44-MG was also detected in eight *Gambierdiscus* species (*G. australes*, *G. caribaeus*, *G. carpenteri*, *G. honu*, *G. jejuensis*, *G. lapillus*, *G. lewisii* and *G. pacificus*) but was not detected in the *Coolia* or *Fukuyoa* species. There were also no detections of the dianhydrogambierone and dianhydro-44-MG analogues (refer to Section 2.6). 29-MG [70] was detected in seven *Gambierdiscus* species (*G. caribaeus*, *G. honu*, *G. jejuensis*, *G. lapillus*, *G. pacificus*, *G. polynesiensis* and *G. scabrosus*), *C. malayensis* CAWD154 and CAWD175, *C. tropicalis* CAWD384 and CAWD388 and *F. paulensis* CAWD238 and CAWD306. 12,13-Dihydro-44-MG was detected in thirteen *Gambierdiscus*, two *Coolia* and two *Fukuyoa* species (the same isolates that produce 44-MG). Due to the extremely close elution time of this analogue and 44-MG, as well as the 2 Da mass difference, there is potential for this to be a false detection. 38-Deoxy-12,13-dihydro-44-MG was detected in eleven *Gambierdiscus* species (*G. belizeanus*, *G. carpenteri*, *G. cheloniae*, *G. holmesii*, *G. honu*, *G. jejuensis*, *G. lapillus*, *G. lewisii*, *G. pacificus*, *G. polynesiensis* and *G. scabrosus*), *C. malayensis* CAWD154 and CAWD175, *C. tropicalis* CAWD384 and CAWD388, *F. paulensis* CAWD238 and CAWD306 and *F. ruetzleri* S044 and S051. Dihydro-sulfo-gambierone was detected in *G. lapillus* CAWD336 and CAWD338, with no sulfo-gambierone being detected.

### 2.4. Other Metabolites

The production of gambieroxide, gambierol and gambieric acids A–D was also investigated. However, in the absence of reference material or positive culture extracts for these metabolites, published MRM transitions were used and results are expressed as detected or not detected (Table 2 and Table S5). A peak corresponding to gambieroxide (eluting at 2.32 min) was observed in *G. lewisii* CAWD369 and *G. pacificus* CAWD227 and CAWD337. Gambieric acid A was the most abundant analogue (eluting at 3.01 min) and was detected in ten *Gambierdiscus* species (*G. australes*, *G. belizeanus*, *G. caribaeus*, *G. carpenteri*, *G. cheloniae*, *G. holmesii*, *G. lapillus*, *G. lewisii*, *G. pacificus* and *G. polynesiensis),* with gambieric acid B (eluting at 3.07 min) being detected in four (*G. australes*, *G. belizeanus*, *G. honu* and *G. jejuensis*). Gambieric acids C and D (eluting at 1.78 and 1.80 min, respectively) were only detected in the *G. australes* CAWD381 isolate. There were no detections of these six metabolites in the *Coolia* or *Fukuyoa* isolates.

### 2.5. Metabolite Spiking Experiments

To give confidence in the results and assess the performance of the analytical methods used, an extract from a representative isolate from each genus (*G. caribaeus* CAWD301, *C. malayensis* CAWD154 and *F. paulensis* CAWD238) was spiked with P-CTX3B, P-CTX3C, P-CTX4A, P-CTX1B, MTX-1, MTX-6, MTX-7, gambierone and 44-MG (Table S6). Each metabolite was spiked at two concentrations, which were adjusted based on the toxin class. Recoveries for the P-CTXs ranged from 91–112%, with those for *G. caribaeus* CAWD301 showing consistently lower results (91–96%). There were no substantial differences between the spiked concentration and recovery of the P-CTXs. The MTXs showed a greater variation in spike recoveries (78–136%), with MTX-7 having the greatest enhancement in the *C. malayensis* CAWD154 isolate (136%). There was no pattern between the suppression/enhancement effects of an individual MTX analogue and the spiked concentration. The recovery of gambierone was enhanced (112–131%), while 44-MG was heavily suppressed (53–66%), although this observation was likely related to interference from the high endogenous levels of this metabolite in the extracts.

Due to the variability observed between the different cultures and spiked metabolites, the results reported herein have not been adjusted and are reported as quantified from the analytical system.

### 2.6. Liquid Chromatography–Mass Spectrometry Analysis of Gambierones

**Figure 8:**
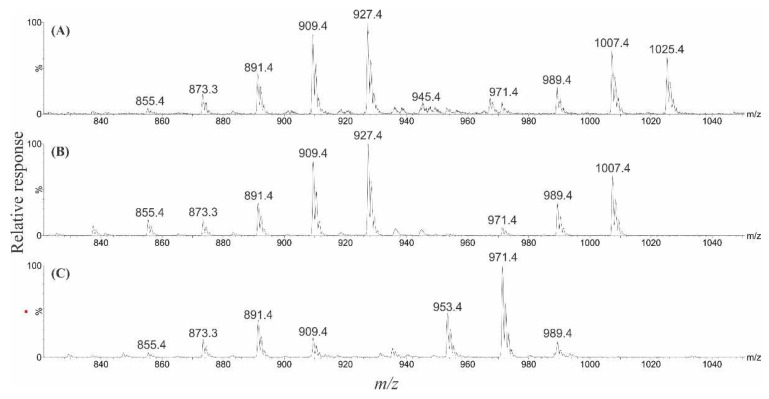
Full scan +ESI mass spectra (displaying *m*/*z* 850–1050) showing the [M + H]^+^, sequential [M + H − *n*H_2_O]^+^, and a series of [M + H − SO_3_ − *n*H_2_O]^+^ ions, of (**A**) gambierone [M + H]^+^ *m*/*z* 1025.4, eluting at 3.56 min, (**B**) anhydrogambierone [M + H]^+^ *m*/*z* 1007.4, eluting at 3.63 min, and (**C**) dianhydrogambierone [M + H]^+^ *m*/*z* 989.4, eluting at 3.85 min.

**Figure 9:**
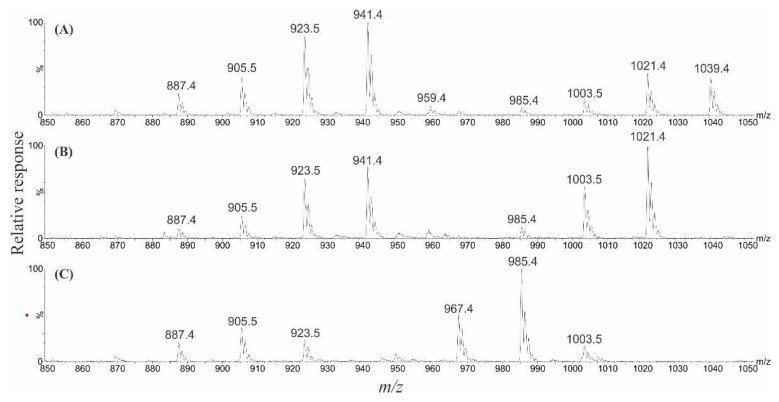
Full scan +ESI mass spectra (displaying *m*/*z* 850–1050) showing the [M + H]^+^, sequential [M + H − *n*H_2_O]^+^, and a series of [M + H − SO_3_ − *n*H_2_O]^+^ ions, of (**A**) 44-MG [M + H]^+^ *m*/*z* 1039.4, eluting at 3.65 min, (**B**) 38-deoxy-44-MG [M + H]^+^ *m*/*z* 1021.4, eluting at 3.75 min, and (**C**) dianhydro-44-MG [M + H]^+^ *m*/*z* 1003.5, eluting at 3.95 min.

LC–MS analysis, both scanning (*m*/*z* 800–1500) and collision induced dissociation (CID) experiments (*m*/*z* 50–1050; varied collision energies (CEs)), were performed on the various fractions generated during the two preparative HPLC fractionations, with four ‘unknown’ gambierone analogues identified. Two had dominant [M + H]^+^ ions of *m*/*z* 1007.4 and 989.4, pertaining to a difference of 18 and 36 Da compared to the [M + H]^+^ ion of gambierone (*m*/*z* 1025.4). Other masses observed represented the [M + H − H_2_O]^+^ ions, followed by [M + H − SO_3_]^+^ and sequential [M + H − SO_3_ − *n*H_2_O]^+^ ions, all of which displayed a mass difference of either 18 or 36 Da compared to that of gambierone (Figure 8). A similar observation was made with 44-MG; two of the analogues had dominant [M + H]^+^ ions of *m*/*z* 1021.4 and 1003.4, pertaining to a mass difference of 18 and 36 Da compared to that of 44-MG (*m*/*z* 1039.4). The subsequent mass spectral pattern also displayed these mass differences (Figure 9). In −ESI mode, the corresponding [M − H]^−^ ions were also 18 and 36 Da less than the primary analogues (Figures S1 and S2). In addition, there is a common in-source fragment ion, in −ESI mode, observed for both gambierone and 44-MG (*m*/*z* 899.2). Similar ions, representing a difference of 18 Da (*m*/*z* 881.3) and 36 Da (*m*/*z* 863.3) were also observed in the spectra of the new analogues (Figures S1 and S2). Therefore, it was hypothesised that these represent anhydro and dianhydro analogues of gambierone and 44-MG, with the ‘anhydro-44-MG’ analogue likely recently published as 38-deoxy-44-MG [56]. Additional evidence to support this hypothesis was the later elution time for the new analogues compared to gambierone and 44-MG, aligning with these analogues being more lipophilic. The limited quantities, and unforeseen instability of these analogues, meant that no further isolation/structural characterisation work could be carried out.

## 3. Discussion

The targeted metabolite fingerprint for thirteen *Gambierdiscus*, five *Coolia* and two *Fukuyoa* species was mapped, and to investigate the intra-species variation two isolates of each species were analysed where possible (34 isolates were analysed in total). Both quantitative and qualitative LC–MS/MS analysis was performed to assess the production of 56 hydrophilic and lipophilic secondary metabolites that have been reported in the literature, as well as the three novel gambierone analogues tentatively identified herein.

The results confirmed that only the *G. polynesiensis* isolates produced the characterised algal P-CTX metabolites, with the profile of both isolates showing a higher abundance of the Type II analogues (e.g., P-CTX3B). Cell quotas for P-CTXs were all lower, by orders of magnitude, than those observed for the gambierones. For example, P-CTX3B was the most abundant analogue, with cell quotas of 1.1 pg/cell and 0.8 pg/cell, compared to the 44-MG cell quotas of 29 pg/cell and 44 pg/cell, for *G. polynesiensis* CAWD212 and CAWD267, respectively. Earlier eluting isomers of P-CTX3B/C and P-CTX4A/B were also observed. The cell quotas ranged from 1.3 to 7.8 pg/cell, which was between seven and forty-fold more than the primary algal P-CTXs, with the highest value (7.8 pg/cell) for iso-P-CTX3B/C produced by *G. polynesiensis* CAWD267. Comparison of the total algal P-CTX cell quotas (a sum of the four primary analogues) showed that *G. polynesiensis* CAWD212 produced more than CAWD267; 1.4 pg/cell and 1.0 pg/cell, respectively. However, when the two isomers were included, *G. polynesiensis* CAWD267 had the highest cell quota of 10.8 pg/cell compared to 7.8 pg/cell in CAWD212. The stereochemistry and relative toxicity of these isomers is currently unknown, with further research required to ascertain the role they play in CP. There was reference material available for six of the 18 fish P-CTX metabolites investigated, with no detections that aligned with said reference materials (retention time or MRM confirmation ratio). Peaks were, however, observed in many of the MRM transitions, with these metabolites being the focus of future compound discovery efforts. In the absence of reference material, production of the C-CTXs and I-CTXs was assessed using published MRM transitions. Early eluting peaks were observed in all MRM transitions for C-CTX1/2 and C-CTX3/4 in *G. scabrosus*; however, they do not align with expected elution times. Further work on these metabolites is required and planned for the future.

Screening of the *Gambierdiscus* isolates for production of the MTX analogues confirmed that only *G. australes* produced MTX-1, as previously demonstrated via a study that analysed 54 *G. australes* isolates. MTX-6 was exclusively produced by the isolates of *G. cheloniae*, and MTX-7 by the *G. honu* isolates, which are the species these secondary metabolites were described from [53]. Interestingly, the cell quotas for MTX-1, MTX-6 and MTX-7 are comparable across the three species. The absence of reference material for MTX-2, MTX-4 and MTX-5 meant that analysis of these analogues was based on mass spectral properties published by Lewis et al. [48], Pisapia et al. [51] and Estevez et al. [52]. Multiple MRM transitions were used to monitor these analogues, representing the doubly- and triply-charged ions. MTX-5 was detected only in *G. australes*, which is the species from where it was first identified [52]. To enable comparison of the cell quota, it was quantified using MTX-1 and an assumed relative response factor of 1. The relative production rates were 30- and 70-fold lower for MTX-5 compared to MTX-1. MTX-5 was also observed in the MTX-1 reference standard, which was used to confirm the MRM confirmation ratio. A compound related to MTX-6 was observed in *G. pacificus*; however, it was later eluting and had a different MRM confirmation ratio. While a compound related to MTX-7 was observed in *G. caribaeus*, it had the same confirmation ratio yet was later eluting. Again, further research is required on these metabolites.

The production of gambierones was widespread amongst the dinoflagellate species tested. 44-MG was the most abundant analogue and now, with the inclusion of *G. jejuensis* and *G. scabrosus* from this study, is ubiquitous in the thirteen *Gambierdiscus* species tested to date. *Gambierdiscus carpenteri* CAWD364 demonstrated the highest production of 44-MG (441 pg/cell), followed by *G. lapillus* CAWD338 (270 pg/cell) and *G. australes* CAWD149 (259 pg/cell). 44-MG has previously been detected in *C. malayensis* and *C. tropicalis* [57], which was confirmed in this study; however, it was not detected in the additional *Coolia* species assessed. Gambierone was produced by fewer species, with only nine *Gambierdiscus*, one *Coolia* and one *Fukuyoa* species producing detectable levels using the current methodology (LoD = 0.01 pg/cell). The highest producer of gambierone was *G. belizeanus* CCMP401 (540 pg/cell), followed by *G. cheloniae* CAWD236 (358 pg/cell).

A further nine gambierone analogues were also investigated, with six analogues being detected. The most abundant of these analogues was 12,13-dihydro-44-MG, which was detected in all the species that produced 44-MG. However, due to the close elution time and similar mass of these analogues it is possible that this is a false detection. Anhydrogambierone and 38-deoxy-44-MG (anticipated to be anhydro-44-MG), which were identified based on the purified material from this study, were detected in eight *Gambierdiscus* species each, although not all the same species, with anhydrogambierone also detected in one *Coolia* and one *Fukuyoa* species. In all cases, these analogues were detected (with a later elution time) in the same isolates that produce gambierone and 44-MG. Surprisingly, there were no detections of the dianhydro analogues described above. Therefore, it is unclear if these analogues are produced below the LoD (0.01 pg/cell) or if they are artifacts created during the isolation process. Due to the very low quantities that were isolated and unexpected instability, no further research was conducted. Conflicting evidence to the hypothesised false detections above, 38-deoxy-12,13-dihydro-44-MG was identified in ten *Gambierdiscus* species, two *Coolia* and two *Fukuyoa* species, some of which did not produce detectable levels of 38-deoxy-44-MG. 29-MG, which is slightly later eluting than 44-MG [70], was detected in six *Gambierdiscus*, two *Coolia* and one *Fukuyoa* species. Lastly, dihydro-sulfogambierone, which elutes between gambierone and 44-MG [55], was only detected in the *G. lapillus* isolates.

The isolates of *G. jejuensis* and *G. scabrosus* were each grown in two different media (f/8 and IMK/2), and while the cell density was higher when grown in IMK/2 media, the cell quotas were almost identical for 44-MG production. In contract, gambierone, produced by *G. scabrosus*, showed a cell quota thirty percent higher when grown in f/8 media. Both IMK/2 and f/8 media are frequently used for culturing *Gambierdiscus* isolates [57,69], with the primary difference being the higher concentration of nitrogen and phosphate, and higher nitrogen:phosphate ratio, in IMK/2 medium, which may explain the difference observed in the production of the secondary metabolites. To investigate this hypothesis, it is, therefore, worth comparing the production of these metabolites by different *Gambierdiscus* species grown under a variety of nutrient concentrations/types of media. Most importantly for this study, the metabolite profile (which can only be assessed based on the gambierone analogues) is the same, irrespective of the media the isolate was grown in.

Six additional secondary metabolites reported to be produced by *Gambierdiscus* species (gambieroxide, gambierol and gambieric acids A–D) were also qualitatively assessed based on published MRM transitions. Of these, gambieric acid A was the most frequently detected (produced by ten *Gambierdiscus* species), while *G. australes* CAWD381 was the only isolate to produce gambieric acids A–D; this is in contrast to the *G. australes* CAWD149 isolate, where only gambieric acids A and B were detected. Gambieric acid B was also detected in three additional species, *G. belizeanus*, *G. honu* (only the CAWD250 isolate) and *G. jejuensis*, whilst neither gambieric acids C nor D were detected in any other species. A peak corresponding to gambieroxide, eluting in the hydrophilic portion of the analysis, was observed in the *G. lewisii* and *G. pacificus* isolates. Gambierol was not detected in the *Gambierdiscus* species, nor were any of these six metabolites detected in the *Coolia* or *Fukuyoa* species. However, in the absence of reference material, and a different analytical system compared to the original research, these results are tentative only.

The spiking experiments demonstrated that the co-extractives and/or high endogenous levels of the spiked metabolite caused both suppression and enhancement effects under LC–MS/MS analysis. Of these metabolites, the P-CTXs were the least impacted, while the MTXs had the greatest variation and 44-MG was heavily suppressed, albeit consistent across the microalgal extracts. The nine metabolites were also spiked at two levels and showed a linear response (either suppression or enhancement).

Deciphering the exact biosynthetic pathway for these secondary metabolites is extremely complex due to the diverse range of genetic features observed in the *Gambierdiscus* genome [71]. However, the polyketide origin of cyclic polyethers suggests that polyketide synthases (PKS) are involved [72]. Gene catalogues for *G. polynesiensis* and *G. excentricus*, two highly toxic species, showed substantial diversity of the PKS genes detected, and a clear distinction was made between those responsible for polyketide biosynthesis and for fatty acid production [72,73]. The array of PKS gene clusters observed suggests that *Gambierdiscus* species are likely to also produce a diverse range of unknown PKS related metabolites, as demonstrated by the numerous ‘unknown’ cyclic polyethers observed in the species investigated [71]. Therefore, the data on contrasting and similar metabolite profiles in the current study will prove useful for researchers working to unravel these genetic pathways, guiding their species selection for further sequencing investigations.

By coupling the metabolite fingerprint of each species, with the limited toxicity information available for the isolates (Table 3) [69] and individual metabolites (Tables S7 and S8), it is clear there is toxicity present that cannot be accounted for based on the known metabolites observed. These isolates will be the focus of future compound discovery efforts.

**Figure 10:**
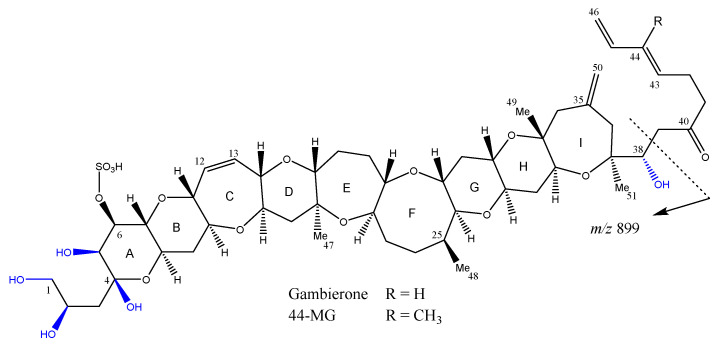
Chemical structure of gambierone and 44-methylgambierone (44-MG) showing the location where in-source fragmentation occurs between C-38 and C-39 (*m*/*z* 899) and the five possible locations for the anhydro and dianhydro variations to occur (labelled in blue).

When undertaking research on *G. honu* CAWD242 (isolation of MTX-7 [53]), several novel gambierone analogues were identified and a series of experiments were performed to determine their likely chemical structures. This involved an array of LC–MS experiments, including full scans in both +ESI and −ESI mode, CID with various CEs and parent ion scans. Interpretation of the results, with comparison to the published spectra for gambierone and 44-MG, provided solid evidence that they were gambierone-like compounds. Two were gambierone analogues with mass differences of 18 and 36 Da less, and two were 44-MG analogues, also with mass differences of 18 and 36 Da less. It was hypothesized that the 18 and 36 Da mass differences observed in these four analogues represented one (anhydro) or two (dianhydro) dehydrations, respectively. A key chromatographic observation to support this hypothesis was the later elution time for the new analogues compared to gambierone and 44-MG. This could be explained by fewer hydroxyl groups, reducing the hydrophilicity of the compounds, thereby increasing their affinity to the hydrophobic stationary phase. Based on the chemical structures of gambierone and 44-MG, it can be determined that the in-source fragment ion observed in −ESI mode (*m*/*z* 899.2) occurs between C-38 and C-39 (Figure 10). The anhydro and dianhydro analogues also had an in-source fragment ion in –ESI mode, which gave ions 18 or 36 Da lower, respectively. There are five hydroxyl groups, that are potential sites for dehydration, between C-1 and C-38 (labelled in blue; Figure 10). In 2022, Liu et al. published 38-deoxy-44-MG [56], and it is anticipated that this is the same analogue as anhydro-44-MG. If correct, it would stand to reason that anhydrogambierone is likely 38-deoxygambierone. The second dehydration, if these are naturally occurring and not an artifact created during the isolation, could happen at four locations. It was hypothesized that the dehydration would likely occur at C-1 or C-2 due to the hydroxyl groups on C-4 and C-5 being shielded by the electron density of the cyclic-ether ring A. However, semisynthetic experiments such as periodate oxidation and/or NMR spectroscopy would be required to determine this, which was not possible due to the low quantities and instability of these metabolites.

In summary, the research presented herein is the most comprehensive targeted metabolite fingerprint performed on the benthic dinoflagellate genera *Gambierdiscus*, *Fukuyoa* and *Coolia* to date. A unique characteristic observed with the *Gambierdiscus* genus is that only a single species produces the algal P-CTXs metabolites, and there is predominately a single MTX analogue produced, which is specific to a particular species. These findings contrast with what is observed with other toxin classes, where multiple species within a genus produce the bioactive secondary metabolites. Furthermore, all the P-CTX- and MTX-producing species were present in Rarotonga, highlighting the significant problem that CP poses in the Cook Islands.

Several *Gambierdiscus* species of interest were identified as they produced a variety of potentially CP-relevant ‘unknown’ secondary metabolites and will be the focus of future compound discovery efforts using bioassay guided fractionation and HR-MS. Lastly, there are many similarities between the polycyclic backbones of the CTXs and gambierones, with a key difference being that the latter is sulphated (for most of the analogues). As described in the preceding paragraphs, gambierones are more prevalent and abundant than CTXs, although they are orders of magnitude lower in toxicity. The question is then raised if they are being biotransformed, as observed for the CTXs, in marine organisms (e.g., desulphated) and play a yet unknown role in CP events.

## 4. Materials and Methods

### 4.1. Mapping the Metabolite Fingerprint

#### 4.1.1. Microalgal Isolates

*Gambierdiscus* isolates (*n* = 9) were cultured (1 L) in either f/8 or IMK/2 media (UV-treated and filtered down to 0.22 µm). The culturing cabinet was set at 25 °C (±2 °C) and had 40–70 µmol·m^−2^·s^−1^ photon irradiance (12:12 h light/dark cycle). Isolates were sourced from previous research expeditions, the Cawthron Institute Culture Collection of Microalgae (Nelson, New Zealand) and Kochi University (Kochi, Japan). Cultures were harvested by centrifugation (1500× *g*, 10 °C, 10 min) in the late exponential or stationary phase. The resulting cell pellets were extracted (sonication aided; 10 min at 59 kHz) with 90% aq. MeOH, at a ratio of 1 mL per 2 × 10^5^ cells. Cellular debris was pelleted via centrifugation (3200× *g*, 10 °C, 10 min), the supernatant decanted and the pellets extracted a second time, giving a final concentration of 1 mL per 1 × 10^5^ cells. Extracellular co-extractives were precipitated in the freezer (−20 °C) and the extract was clarified using centrifugation.

Existing extracts were used for the remaining *Gambierdiscus* (*n* = 12), *Coolia* (*n* = 9) and *Fukuyoa* (*n* = 4) isolates [23,57].

#### 4.1.2. Targeted Liquid Chromatography–Mass Spectrometry

Analyses were performed on a Waters Xevo TQ-S triple quadrupole mass spectrometer coupled to a Waters Acquity UPLC i-Class with flow-through needle sample manager. The mass spectrometer utilised ESI (positive and negative ion modes), and chromatographic separation was achieved on a Waters Acquity UPLC BEH phenyl column (1.7 μm, 100 × 2.1 mm) held at 50 °C. The column was eluted using mobile phases containing 0.2% (*v*/*v*) of a 25% NH_4_OH solution in (A) Milli Q water and (B) 95% aqueous MeCN, with the flow rate used for each method specified below. Fresh mobile phases were prepared daily to ensure optimal sensitivity and stable retention times. The autosampler chamber was maintained at 10 °C and the injection volume was 2 μL for CTXs and 1 µL for the other metabolite classes. Other settings were capillary voltage 3.0 kV, cone voltage 40 V, source temperature 150 °C, N_2_ gas desolvation flow rate 1000 L/h at 600 °C, cone gas 150 L/h, and the collision cell was operated with 0.15 mL/min argon. Data acquisition and processing were performed with MassLynx and TargetLynx software, respectively. MRM transitions were either experimentally determined using reference material (purified compounds, algal and fish extracts) found in the literature, or calculated using ChemDraw based on known fragmentation patterns and structural modifications. A full list of the MRM transitions for the 56 hydrophilic and lipophilic metabolites can be found in Supplementary Tables S9–S14.

##### Ciguatoxins

The initial solvent composition was 5% B, with a linear gradient to 50% B from 1 to 3.5 min, a linear gradient to 75% B from 3.5 to 7.5 min, ramped up to 95% B by 8 min and held at 95% B until 9 min, followed by a linear gradient back to 5% B at 9.2 min. The column was then re-equilibrated with 5% B for 0.8 min. The total injection time was 10 min, with a flow rate of 0.55 mL/min.

Quantitative analysis was performed using five-point linear regression calibration curves (*R*^2^ > 0.98) of a mixed qNMR P-CTX standard (P-CTX3B, P-CTX3C, P-CTX4A and P-CTX1B; linear range of 0.5–10 ng/mL) that was forced through zero. The calibration curves for P-CTX3B and P-CTX4A were used to quantify iso-P-CTX3B/C and P-CTX4B and iso-P-CTX4A/B, respectively, with a relative response factor of 1 (elution order of the algal P-CTX metabolites is shown in Figure S3). Reference materials (qNMR standards or ciguatoxic fish extracts) were used to confirm the elution time and confirmation ratio between MRM transitions for 52-*epi*-54-deoxy-CTX1B, 54-deoxy-CTX1B, 51-hydroxy-CTX3C, 2,3-dihydroxy-CTX3B and 2,3-dihydroxy-CTX3C.

##### Maitotoxins and Gambieroxide

The initial solvent composition was 5% B, with a linear gradient to 30% B from 0.5 to 1 min, a linear gradient to 50% B from 1 to 3 min, ramped up to 95% B by 3.5 min and held at 95% B until 3.7 min, followed by a linear gradient back to 5% B at 4 min. The column was then re-equilibrated with 5% B for 0.5 min. The total injection time was 4.5 min with a flow rate of 0.5 mL/min.

The MTXs were quantified using a six-point linear regression calibration curve (*R*^2^ > 0.98) of a certified MTX-1 standard (2.5–200 ng/mL) that was forced through zero. A relative response factor was used to quantify MTX-5, MTX-6 and MTX-7. Purified MTX-6 and MTX-7 material was used to confirm the retention time and confirmation ratio between MRM transitions. Detection of gambieroxide was qualitative only as no standard was available (elution order of the MTXs and gambieroxide metabolites is shown in Figure S4).

##### Gambierones

The flow rate was 0.55 mL/min, with an initial solvent composition of 5% B, a linear gradient to 50% B from 1 to 2.5 min, ramped up to 95% B by 3 min and held at 95% B until 3.2 min, followed by a linear gradient back to 5% B at 3.5 min. The column was then re-equilibrated with 5% B for 0.5 min. The total injection time was 4 min.

Quantitative analysis was performed using a six-point linear regression calibration curve (*R*^2^ > 0.98) of an in-house qNMR certified 44-MG standard (linear range of 5–1000 ng/mL) that was forced through zero. An experimentally determined relative response factor of 1 was used to quantify the gambierone cell quotas. Purified gambierone material was used to confirm the retention time and confirmation ratio between MRM transitions. The remaining gambierone analogues were qualitatively assessed (elution order of the gambierone metabolites is shown in Figure S5).

##### Gambierol and Gambieric Acids

The initial solvent composition was 5% B, with a linear gradient to 95% B from 0.5 to 3.8 min, held at 95% B until 4.2 min, followed by a linear gradient back to 5% B at 4.5 min. The column was then re-equilibrated with 5% B for 0.5 min. The total injection time was 5 min.

Detection of gambierol and gambieric acids A–D was qualitative only as no standards were available (elution order of the gambieric acid metabolites is shown in Figure S6).

### 4.2. Tentative Gambierone Analogues

#### 4.2.1. Culturing

The *G. honu* CAWD242 isolate was grown in f/2 seawater (1:3; UV-treated and filtered down to 0.22 µm). The culturing cabinet had 40–70 µmol·m^−2^·s^−1^ photon irradiance (12:12 h light/dark cycle) and was set at 25 °C (±2 °C). Consecutive 5 L monoclonal cultures (total of 100 L) were grown to produce enough biomass (1.8 × 10^8^ cells) and harvested during the stationary phase of the growth cycle by centrifugation (3200× *g*, 10 °C, 10 min). The resulting cell pellets were frozen (−20 °C) until ready for extraction. This biomass was primarily generated for the isolation of MTX-7 [53].

#### 4.2.2. Extraction and Isolation

The extraction and isolation procedure is described in Murray et al., 2022 [53]. In brief, the cell pellets underwent a triple extraction with 90% aq. MeOH (sonication aided). The supernatant was frozen (−20 °C) to precipitate extracellular co-extractives and clarified using sequential glass fiber and membrane filtration (8 µm, 2 µm and 1.6 µm). Lipids were removed via a liquid–liquid partition with *n*-hexane (1:1, *v*/*v*); the extract was then diluted to 60% aq. MeOH and a second partition with dichloromethane (DCM; 1:1, *v*/*v*) was performed to separate the hydrophilic and lipophilic metabolites. The aq. MeOH layer was dried under rotary evaporation (50 mBar/50 °C) and redissolved in acidified Milli-Q. The extract was loaded onto a Strata-X solid phase extraction column (10 g) and washed with 60% aq. MeOH; the gambierones were eluted with 100% MeOH, dried under rotary evaporation (50 mBar/50 °C) and redissolved in 20% aq. MeOH. The extract was then fractionated on a Reveleris flash chromatography system fitted with an Agilent Superflash C_18_ SF 25–75 g column, and two wavelengths (210 and 230 nm) were monitored. The column was eluted (20 mL/min) with (A) Milli-Q water and (B) MeCN mobile phases, both with the addition of 0.05% acetic acid (*v*/*v*). The fractions of interest were then subjected to two further purification steps using a Shimadzu preparative HPLC-PDA system with a Phenomenex Gemini C_18_ column (5 µm; 150 × 21.2 mm) and UV detection (190–300 nm). The column was eluted isocratically (25 mL/min) with 38% and then 35% aq. MeCN mobile phase containing 0.2% (*v*/*v*) of a 25% NH_4_OH solution.

Six gambierone analogues were isolated: four in the first fractionation on the Shimadzu instrument (gambierone, anhydro-44-MG (38-deoxy-44-MG), dianhydrogambierone and dianhydro-44-MG) in fractions 2, 4, 9 and 12, respectively, and two in the second fractionation (anhydrogambierone and 44-MG) in fractions 4 and 6, respectively (Figure S3).

#### 4.2.3. Liquid Chromatography–Mass Spectrometry Analysis of Gambierone Analogues

The gambierone analogues were analysed on a Waters Xevo TQ-S triple quadrupole mass spectrometer coupled to a Waters Acquity UPLC i-Class with a flow-through needle sample manager. Scanning experiments (*m*/*z* 200–1200) were performed in positive and negative electrospray ionization (ESI) modes, and chromatographic separation was achieved on a Waters Acquity UPLC BEH phenyl column (1.7 μm, 100 × 2.1 mm) held at 50 °C. Mobile phases containing 0.2% (*v*/*v*) of a 25% NH_4_OH solution in (A) Milli-Q water and (B) MeCN were used to elute the column with a flow rate of 0.55 mL/min. Initial solvent conditions were 5% B for 1 min with a linear gradient to 95% B from 1.0 to 7.5 min, held at 95% B for 1 min, followed by a linear gradient back to 5% B from 8.5 to 9 min. The column was re-equilibrated with 5% B until 10 min. Fresh mobile phases were prepared daily to ensure optimal sensitivity and stable retention times. The autosampler chamber was maintained at 10 °C and the injection volume was 1 μL.

## 5. Conclusions

The targeted metabolite fingerprints, CP focused, were mapped for 34 benthic microalgal isolates consisting of thirteen *Gambierdiscus*, five *Coolia* and two *Fukuyoa* species. Of the secondary metabolites analysed, gambierones were the most widely produced, with detections in isolates from the three genera of benthic microalgae. Only species of *Gambierdiscus* produced CTXs, MTXs, gambieroxide or gambieric acids, illustrating that this genus is the primary cause of CP—based on the known bioactive metabolites. In addition, four tentative gambierone analogues were isolated and hypothesized to represent anhydro and dianhydro variants of gambierone and 44-MG. To date, this is the most comprehensive secondary metabolite mapping performed on these genera in a single study, highlighting several species with unaccounted toxicity, and therefore a high probability of novel bioactive metabolites still to be discovered.

## Figures and Tables

**Table 1:** Summary of the metabolite cell quotas in 34 benthic dinoflagellate isolates from thirteen *Gambierdiscus*, five *Coolia* and two *Fukuyoa* species.

Culture ID	Scientific Name	Location	pg/Cell											
			P-CTX				iso-P-CTX		MTX				G	44-MG
			3B	3C	4A	4B *^a^*	3B/C *^b^*	4A/B *^a^*	−1	−5 *^c,^^d^*	−6 *^d^*	−7 *^d^*		
CAWD149	*G. australes*	Rarotonga	◦	◦	◦	◦	◦	◦	6	0.2	◦	◦	◦ *^e^*	259 *^e^*
CAWD381	*G. australes*	Kermadec Islands	◦	◦	◦	◦	◦	◦	9	0.1	◦	◦	◦	160
CCMP401	*G. belizeanus*	St. Barthelemy Island	◦	◦	◦	◦	◦	◦	◦	◦	◦	◦	540	53
CAWD301	*G. caribaeus*	Pohnpei	◦	◦	◦	◦	◦	◦	◦	◦	◦	◦	◦ *^e^*	44 *^e^*
CAWD237	*G. carpenteri* *^f^*	Australia	◦	◦	◦	◦	◦	◦	◦	◦	◦	◦	87 *^e^*	74 *^e^*
CAWD237	*G. carpenteri*	Australia	◦	◦	◦	◦	◦	◦	◦	◦	◦	◦	65 *^e^*	45 *^e^*
CAWD364	*G. carpenteri*	Australia	◦	◦	◦	◦	◦	◦	◦	◦	◦	◦	◦	441
CAWD232	*G. cheloniae*	Rarotonga	◦	◦	◦	◦	◦	◦	◦	◦	4	◦	55 *^e^*	26 *^e^*
CAWD236	*G. cheloniae*	Rarotonga	◦	◦	◦	◦	◦	◦	◦	◦	5	◦	358	43
CAWD368	*G. holmesii*	Australia	◦	◦	◦	◦	◦	◦	◦	◦	◦	◦	20 *^e^*	97 *^e^*
CAWD242	*G. honu*	Rarotonga	◦	◦	◦	◦	◦	◦	◦	◦	◦	14	38 *^e^*	182 *^e^*
CAWD250	*G. honu*	Tonga	◦	◦	◦	◦	◦	◦	◦	◦	◦	2	42	54
NIES-4120	*G. jejuensis*	Japan	◦	◦	◦	◦	◦	◦	◦	◦	◦	◦	◦	71
NIES-4120	*G. jejuensis* *^g^*	Japan	◦	◦	◦	◦	◦	◦	◦	◦	◦	◦	◦	72
CAWD336	*G. lapillus*	Rarotonga	◦	◦	◦	◦	◦	◦	◦	◦	◦	◦	◦ *^e^*	46 *^e^*
CAWD338	*G. lapillus*	Rarotonga	◦	◦	◦	◦	◦	◦	◦	◦	◦	◦	◦ *^e^*	270 *^e^*
CAWD369	*G. lewisii*	Australia	◦	◦	◦	◦	◦	◦	◦	◦	◦	◦	1 *^e^*	68 *^e^*
CAWD227	*G. pacificus*	Rarotonga	◦	◦	◦	◦	◦	◦	◦	◦	◦	◦	8	65
CAWD337	*G. pacificus*	Rarotonga	◦	◦	◦	◦	◦	◦	◦	◦	◦	◦	1 *^e^*	100 *^e^*
CAWD212	*G. polynesiensis*	Rarotonga	1.1	0.19	0.13	0.04	5.1	1.3	◦	◦	◦	◦	13 *^e^*	29 *^e^*
CAWD267	*G. polynesiensis*	Rarotonga	0.82	0.14	0.05	0.02	7.8	2.0	◦	◦	◦	◦	13 *^e^*	44 *^e^*
CAWD429	*G. scabrosus*	Japan	◦	◦	◦	◦	◦	◦	◦	◦	◦	◦	131	17
CAWD429	*G. scabrosus* *^g^*	Japan	◦	◦	◦	◦	◦	◦	◦	◦	◦	◦	102	17
K070922_1	*G. scabrosus*	Japan	◦	◦	◦	◦	◦	◦	◦	◦	◦	◦	166	32
CAWD385	*C. canariensis*	Kermadec Islands	◦	◦	◦	◦	◦	◦	◦	◦	◦	◦	◦	◦
CAWD387	*C. canariensis*	Kermadec Islands	◦	◦	◦	◦	◦	◦	◦	◦	◦	◦	◦	◦
CAWD154	*C. malayensis*	New Zealand	◦	◦	◦	◦	◦	◦	◦	◦	◦	◦	2 *^e^*	9 *^e^*
CAWD175	*C. malayensis*	New Zealand	◦	◦	◦	◦	◦	◦	◦	◦	◦	◦	17 *^e^*	24 *^e^*
CAWD60	*C. monotis*	Spain	◦	◦	◦	◦	◦	◦	◦	◦	◦	◦	◦	◦
UTS4	*C. palmyrensis*	Australia	◦	◦	◦	◦	◦	◦	◦	◦	◦	◦	◦	◦
UTS25	*C. palmyrensis*	Australia	◦	◦	◦	◦	◦	◦	◦	◦	◦	◦	◦	◦
CAWD384	*C. tropicalis*	Kermadec Islands	◦	◦	◦	◦	◦	◦	◦	◦	◦	◦	◦ *^e^*	14 *^e^*
CAWD388	*C. tropicalis*	Kermadec Islands	◦	◦	◦	◦	◦	◦	◦	◦	◦	◦	◦ *^e^*	15 *^e^*
CAWD238	*F. paulensis*	New Zealand	◦	◦	◦	◦	◦	◦	◦	◦	◦	◦	◦ *^e^*	5 *^e^*
CAWD306	*F. paulensis*	New Zealand	◦	◦	◦	◦	◦	◦	◦	◦	◦	◦	◦ *^e^*	65 *^e^*
S044	*F. ruetzleri*	Hong Kong	◦	◦	◦	◦	◦	◦	◦	◦	◦	◦	8 *^e^*	12 *^e^*
S051	*F. ruetzleri*	Hong Kong	◦	◦	◦	◦	◦	◦	◦	◦	◦	◦	6 *^e^*	13 *^e^*

*G*. = *Gambierdiscus*, *C*. = *Coolia*, *F*. = *Fukuyoa*, CTX = ciguatoxin, MTX = maitotoxin, G = gambierone, 44-MG = 44-methylgambierone, ◦ = <0.01 pg/cell. All isolates were grown in f/2 media, unless marked with a specific footnote. *^a^*Quantified using an LC–MS/MS calibrated reference standard of P-CTX4A, with a relative response factor of 1. *^b^* Quantified using an LC–MS/MS calibrated reference standard of P-CTX3B, with a relative response factor of 1. *^c^* Analysis was performed using published MRM transitions for these compounds and detections are tentative only until reference material is available. *^d^* Quantified using an LC–MS/MS calibrated reference standard of MTX-1, with a relative response factor of 1. *^e^* Results reported in Murray et al., 2021 [23]. *^f^* Grown in K media. *^g^* Grown in IMK/2 media.

**Table 2:** Summary of metabolite detections in 34 benthic dinoflagellate isolates from thirteen *Gambierdiscus*, five *Coolia* and two *Fukuyoa* species.

Culture ID	Scientific Name	Location	Detected/Not Detected										
			Anhydro G	DiH-sulfo-G *^a^*	12,13-diH-44-MG *^a^*	29-MG	38-deOH-44-MG *^a^*	38-deOH-12,13-diH-44-MG *^a^*	Gambieroxide *^a^*	Gambieric Acid *^a^*			
										**A**	**B**	**C**	**D**
CAWD149	*G. australes*	Rarotonga	◦	◦	D	◦	D	◦	◦	D	D	◦	◦
CAWD381	*G. australes*	Kermadec Islands	◦	◦	D	◦	D	◦	◦	D	D	D	D
CCMP401	*G. belizeanus*	St. Barthelemy Island	D	◦	D	◦	◦	D	◦	D	D	◦	◦
CAWD301	*G. caribaeus*	Pohnpei	◦	◦	D	D	D	◦	◦	D	◦	◦	◦
CAWD237	*G. carpenteri* *^b^*	Australia	D	◦	D	◦	D	D	◦	D	◦	◦	◦
CAWD237	*G. carpenteri*	Australia	D	◦	D	◦	D	D	◦	D	◦	◦	◦
CAWD364	*G. carpenteri*	Australia	◦	◦	D	◦	◦	D	◦	D	◦	◦	◦
CAWD232	*G. cheloniae*	Rarotonga	D	◦	D	◦	◦	D	◦	D	◦	◦	◦
CAWD236	*G. cheloniae*	Rarotonga	D	◦	D	◦	◦	D	◦	◦	◦	◦	◦
CAWD368	*G. holmesii*	Australia	D	◦	D	◦	◦	D	◦	D	◦	◦	◦
CAWD242	*G. honu*	Rarotonga	D	◦	D	◦	D	D	◦	◦	◦	◦	◦
CAWD250	*G. honu*	Tonga	D	◦	D	D	D	D	◦	◦	D	◦	◦
NIES-4120	*G. jejuensis*	Japan	◦	◦	D	D	D	D	◦	◦	◦	◦	◦
NIES-4120	*G. jejuensis* *^c^*	Japan	◦	◦	D	D	D	D	◦	◦	D	◦	◦
CAWD336	*G. lapillus*	Rarotonga	◦	D	D	D	D	D	◦	D	◦	◦	◦
CAWD338	*G. lapillus*	Rarotonga	◦	D	D	D	D	D	◦	D	◦	◦	◦
CAWD369	*G. lewisii*	Australia	◦	◦	D	◦	D	D	D	D	◦	◦	◦
CAWD227	*G. pacificus*	Rarotonga	D	◦	D	D	D	D	D	D	◦	◦	◦
CAWD337	*G. pacificus*	Rarotonga	D	◦	D	D	D	D	D	D	◦	◦	◦
CAWD212	*G. polynesiensis*	Rarotonga	D	◦	D	D	◦	D	◦	D	◦	◦	◦
CAWD267	*G. polynesiensis*	Rarotonga	D	◦	D	D	◦	D	◦	D	◦	◦	◦
CAWD429	*G. scabrosus*	Japan	D	◦	D	D	◦	D	◦	◦	◦	◦	◦
CAWD429	*G. scabrosus* *^c^*	Japan	D	◦	D	D	◦	D	◦	◦	◦	◦	◦
K070922_1	*G. scabrosus*	Japan	D	◦	D	D	◦	D	◦	◦	◦	◦	◦
CAWD385	*C. canariensis*	Kermadec Islands	◦	◦	◦	◦	◦	◦	◦	◦	◦	◦	◦
CAWD387	*C. canariensis*	Kermadec Islands	◦	◦	◦	◦	◦	◦	◦	◦	◦	◦	◦
CAWD154	*C. malayensis*	New Zealand	D	◦	D	D	◦	D	◦	◦	◦	◦	◦
CAWD175	*C. malayensis*	New Zealand	D	◦	D	D	◦	D	◦	◦	◦	◦	◦
CAWD60	*C. monotis*	Spain	◦	◦	◦	◦	◦	◦	◦	◦	◦	◦	◦
UTS4	*C. palmyrensis*	Australia	◦	◦	◦	◦	◦	◦	◦	◦	◦	◦	◦
UTS25	*C. palmyrensis*	Australia	◦	◦	◦	◦	◦	◦	◦	◦	◦	◦	◦
CAWD384	*C. tropicalis*	Kermadec Islands	◦	◦	D	D	◦	D	◦	◦	◦	◦	◦
CAWD388	*C. tropicalis*	Kermadec Islands	◦	◦	D	D	◦	D	◦	◦	◦	◦	◦
CAWD238	*F. paulensis*	New Zealand	◦	◦	D	D	◦	D	◦	◦	◦	◦	◦
CAWD306	*F. paulensis*	New Zealand	◦	◦	D	D	◦	D	◦	◦	◦	◦	◦
S044	*F. ruetzleri*	Hong Kong	D	◦	D	◦	◦	D	◦	◦	◦	◦	◦
S051	*F. ruetzleri*	Hong Kong	D	◦	D	◦	◦	D	◦	◦	◦	◦	◦

*G.* = *Gambierdiscus*; *C.* = *Coolia*; *F.* = *Fukuyoa*; D = detected; G = gambierone; -MG = -methylgambierone; DiH = dihydro; deOH = deoxygenated; ◦ = not detected (detection limit unknown as reference material was not available). All isolates were grown in f/2 media, unless marked with a specific footnote. *^a^* Analysis was performed using published MRM transitions for these compounds and detections are tentative only until reference material is available. *^b^* Grown in K media. *^c^* Grown in IMK/2 media.

**Table 3:** Summary of metabolite detections in 34 benthic dinoflagellate isolates of thirteen *Gambierdiscus*, five *Coolia* and two *Fukuyoa* species.

Isolates	Scientific Name	Metabolites Detected								Acute Toxicity (LD_50_; mg/kg)		
		CTXs	MTXs	G	44-MG	Other Gs	GOx	GA A-B	GA C-D	Isolate (CAWD)	i.p.	Orally
CAWD149 and CAWD381	*G. australes*		X		X	X		X	X *^b^*	149 *^a^*	0.37	40
CCMP401	*G. belizeanus*			X	X	X		X				
CAWD301	*G. caribaeus*				X	X		X		– *^d^*		
CAWD237 *^c^* and CAWD364	*G. carpenteri*			X *^b^*	X	X		X		237 *^a^*	10	>158
CAWD232 and CAWD236	*G. cheloniae*		X	X	X	X		X		232 *^a^*	0.32	118
CAWD368	*G. holmesii*			X	X	X		X		– *^e^*		
CAWD242 and CAWD250	*G. honu*		X	X	X	X		X		242 *^a^*	0.2	100
NIES-4120 *^c^*	*G. jejuensis*				X	X		X				
CAWD336 and CAWD338	*G. lapillus*				X	X		X		– *^e^*		
CAWD369	*G. lewisii*			X	X	X	X	X		– *^e^*		
CAWD227 and CAWD337	*G. pacificus*			X	X	X	X	X		227 *^a^*	0.4	>400
CAWD212 and CAWD267	*G. polynesiensis*	X		X	X	X		X		212 *^a^*	1.88	3.2
CAWD429 *^c^* and K070922_1	*G. scabrosus*			X	X	X						
CAWD385 and CAWD387	*C. canariensis*									– *^d^*		
CAWD154 and CAWD175	*C. malayensis*			X	X	X				– *^d^*		
CAWD60	*C. monotis*									– *^d^*		
UTS4 and UTS25	*C. palmyrensis*									– *^d^*		
CAWD384 and CAWD388	*C. tropicalis*				X	X				– *^d^*		
CAWD238 and CAWD306	*F. paulensis*				X	X				238 *^a^*	10	>790
S044 and S051	*F. ruetzleri*			X	X	X						

*G*. = *Gambierdiscus*; *C*. = *Coolia*; *F*. = *Fukuyoa*; CTX = ciguatoxin; MTX = maitotoxin; G = gambierone; 44-MG = 44-methygambierone; Other Gs = additional gambierone analogues; GOx = gambieroxide; GA = gambieric acid; i.p. = intraperitoneal injection; – = isolate does not have a CAWD number. *^a^* Published in Munday et al., 2017 [69]. *^b^* Only detected in one isolate. *^c^* Grown in two different media. *^d^* Different isolates of this species (not analysed during this study) are reported to be of low or no toxicity (unpublished). *^e^* Different isolates of this species (not analysed during this study) are reported to be toxic via i.p. injection (unpublished).

## Data Availability

The original contributions presented in the study are included in the article/Supplementary Materials, further inquiries can be directed to the corresponding author.

## Supplementary Materials

The following supporting information can be downloaded at: https://www.mdpi.com/article/10.3390/md22030119/s1. Figure S1. Full scan –ESI mass spectra (displaying *m*/*z* 850–1050) showing the [M − H]^−^ ion and in-source fragment ion of (A) gambierone [M−H]^−^ *m*/*z* 1023.3, eluting at 3.56 min, (B) anhydrogambierone [M − H]^−^ *m*/*z* 1005.3, eluting at 3.63 min, and (C) dianhydrogambierone [M − H]^−^ *m*/*z* 987.3, eluting at 3.85 min; Figure S2. Full scan –ESI mass spectra (displaying *m*/*z* 850–1050) showing the [M − H]^−^ ion and in-source fragment ion of (A) 44-MG [M − H]^−^ *m*/*z* 1037.3, eluting at 3.65 min, (B) anhydro-44-MG [M − H]^−^ *m*/*z* 1019.3, eluting at 3.75 min and (C) dianhydro-44-MG [M − H]^−^ *m*/*z* 1001.3, eluting at 3.95 min; Figure S3. Example chromatogram showing the elution order of the primary P-CTX algal metabolites; Figure S4. Example chromatogram showing the elution order of the MTXs and gambieroxide metabolites; Figure S5. Example chromatogram showing the elution order of the gambierone metabolites; Figure S6. Example chromatogram showing the elution order of the gambieric acid metabolites; Figure S7. Purification scheme of the anhydro and dianhydro gambierone analogues; Table S1. Cell quotas of the algal CTX metabolites produced by 34 isolates representing thirteen *Gambierdiscus*, five *Coolia* and two *Fukuyoa* species; Table S2. Qualitative analysis of C-CTX and I-CTX produced by 34 isolates representing thirteen *Gambierdiscus*, five *Coolia* and two *Fukuyoa* species; Table S3. Cell quotas of maitotoxins produced by 34 isolates representing thirteen *Gambierdiscus*, five *Coolia* and two *Fukuyoa* species; Table S4. Cell quotas and qualitative analysis of gambierones produced by 34 isolates representing *Gambierdiscus*, five *Coolia* and two *Fukuyoa* species; Table S5. Qualitative analysis of six additional metabolites produced by 34 isolates representing *Gambierdiscus*, five *Coolia* and two *Fukuyoa* species; Table S6. Metabolite spike recoveries in representative isolates of *Gambierdiscus, Coolia* and *Fukuyoa* species; Table S7. Summary of the toxicity information (in vivo and in vitro) available for the ciguatoxins and maitotoxins; Table S8. Summary of the toxicity information (in vivo and in vitro) available for the gambierones, gambieroxide, gambierol and gambieric acids; Table S9. List of the MRM transitions and CEs used for the Type I Pacific ciguatoxins monitored; Table S10. List of the MRM transitions and CEs used for the Type II Pacific ciguatoxins monitored; Table S11. List of the MRM transitions and CEs used for the Caribbean and Indian ciguatoxins monitored; Table S12. List of the MRM transitions and CEs used for the maitotoxins monitored; Table S13. List of the MRM transitions and CEs used for the gambierones monitored; Table S14. List of the MRM transitions and CEs used for the gambieroxide, gambierol and the gambieric acids.
