# Supplementary material for: Targeted Metabolite Fingerprints of Thirteen Gambierdiscus, Five Coolia and Two Fukuyoa Species
Source: Mar Drugs. 2024 Mar 2;22(3):119. doi: 10.3390/md22030119 (PMC10972191; doi:10.3390/md22030119)
Supplement: Supplementary file 1 [file marinedrugs-22-00119-s001.zip › marinedrugs-2859763-supplementary.pdf]

Supplementary information for

Targeted Metabolite Fingerprints of Thirteen *Gambierdiscus*, Five *Coolia* and Two *Fukuyoa* Species

J. Sam Murray,* Emillie M.F. Passfield, Lesley L. Rhodes, Jonathan Puddick, Sarah C. Finch, Kirsty F. Smith, Roel van Ginkel, Elizabeth M. Mudge, Tomohiro Nishimura, Hiroshi Funaki, Masao Adachi, Michèle R. Prinsep and D. Tim Harwood

* Corresponding author – sam.murray@cawthron.org.nz

Figure S1. Full scan –ESI mass spectra (displaying *m/z* 850–1,050) showing the [M–H]$^-$ ion and in-source fragment ion of (**A**) gambierone [M–H]$^-$ *m/z* 1,023.3, eluting at 3.56 min, (**B**) anhydrogambierone [M–H]$^-$ *m/z* 1,005.3, eluting at 3.63 min, and (**C**) dianhydrogambierone [M–H]$^-$ *m/z* 987.3, eluting at 3.85 min 4

Figure S2. Full scan –ESI mass spectra (displaying *m/z* 850–1,050) showing the [M–H]$^-$ ion and in-source fragment ion of (**A**) 44-MG [M–H]$^-$ *m/z* 1,037.3, eluting at 3.65 min, (**B**) anhydro-44-MG [M–H]$^-$ *m/z* 1,019.3, eluting at 3.75 min, and (**C**) dianhydro-44-MG [M–H]$^-$ *m/z* 1,001.3, eluting at 3.95 min 4

Figure S3. Example chromatogram showing the elution order of the primary P-CTX algal metabolites 24

Figure S4. Example chromatogram showing the elution order of the MTXs and gambieroxide metabolites 24

Figure S5. Example chromatogram showing the elution order of the gambierone metabolites.......................... 25

Figure S6. Example chromatogram showing the elution order of the gambieric acid metabolites...................... 25

Figure S7. Purification scheme of the anhydro and dianhydro gambierone analogues 26

Table S1. Cell quotas of the algal CTX metabolites produced by 34 isolates representing 13 *Gambierdiscus*, five *Coolia* and two *Fukuyoa* species 5

Table S2. Qualitative analysis of C-CTX and I-CTX produced by 34 isolates representing 13 *Gambierdiscus*, five *Coolia* and two *Fukuyoa* species 7

Table S3. Cell quotas of maitotoxins produced by 34 isolates representing 13 *Gambierdiscus*, five *Coolia* and two *Fukuyoa* species 9

Table S4. Cell quotas and qualitative analysis of gambierones produced by 34 isolates representing *Gambierdiscus*, five *Coolia* and two *Fukuyoa* species 11

Table S5. Qualitative analysis of six additional metabolites produced by 34 isolates representing *Gambierdiscus*, five *Coolia* and two *Fukuyoa* species 13

Table S6. Metabolite spike recoveries in representative isolates of *Gambierdiscus, Coolia* and *Fukuyoa* species...15

Table S7. Summary of the toxicity information (*in vivo* and *in vitro*) available for the ciguatoxins and maitotoxins 16

Table S8. Summary of the toxicity information (*in vivo* and *in vitro*) available for the gambierones, gambieroxide, gambierol and the gambieric acids 17

Table S9. List of the MRM transitions and CEs used for the Type I Pacific ciguatoxins monitored 18

Table S10. List of the MRM transitions and CEs used for the Type II Pacific ciguatoxins monitored 19

Table S11. List of the MRM transitions and CEs used for the Caribbean and Indian ciguatoxins monitored 20

Table S12. List of the MRM transitions and CEs used for the maitotoxins monitored 21

Table S13. List of the MRM transitions and CEs used for the gambierones monitored 22

Table S14. List of the MRM transitions and CEs used for the gambieroxide, gambierol and the gambieric acids 23

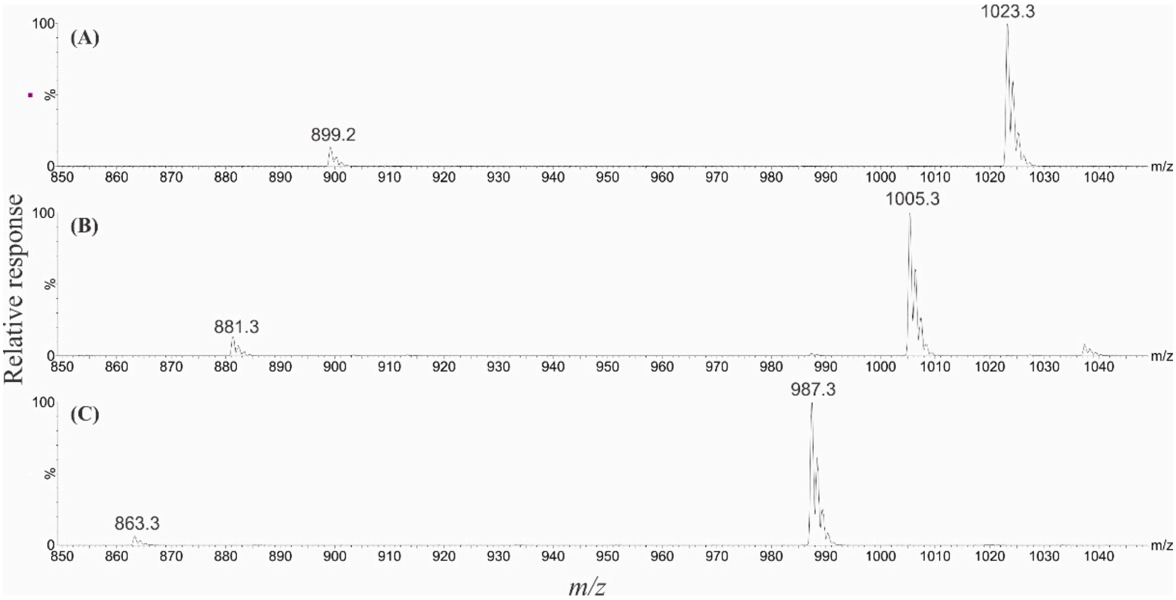

Figure S1. Full scan –ESI mass spectra (displaying *m/z* 850–1,050) showing the [M–H]- ion and in-source fragment ion of (**A**) gambierone [M–H]- *m/z* 1,023.3, eluting at 3.56 min, (**B**) anhydrogambierone [M–H]- *m/z* 1,005.3, eluting at 3.63 min, and (**C**) dianhydrogambierone [M–H]- *m/z* 987.3, eluting at 3.85 min.

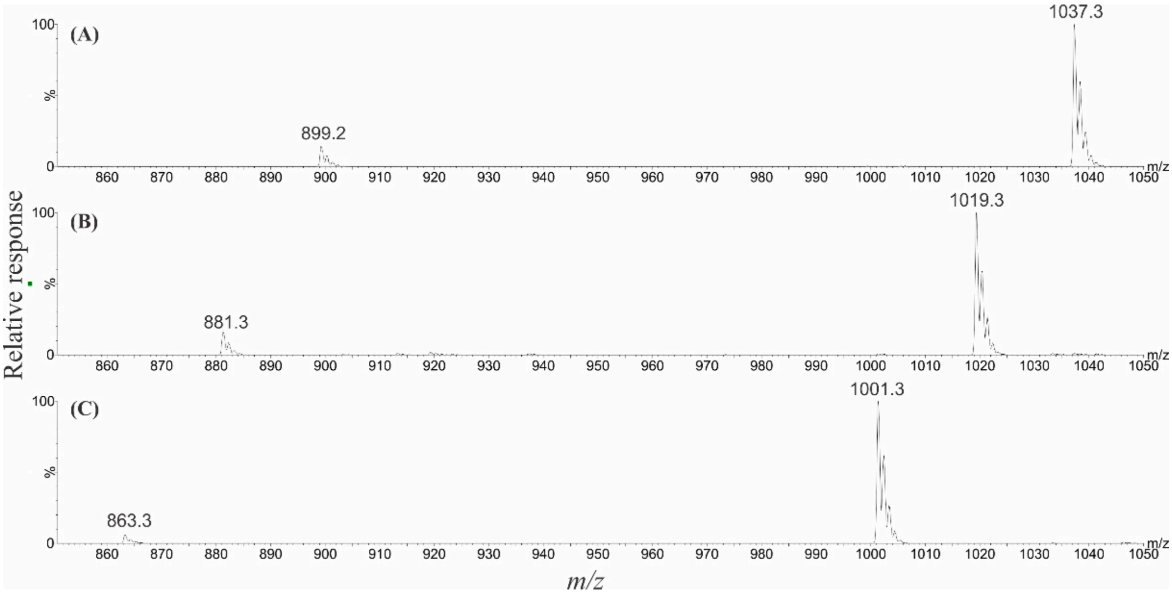

Figure S2. Full scan –ESI mass spectra (displaying *m/z* 850–1,050) showing the [M–H]- ion and in-source fragment ion of (**A**) 44-MG [M–H]- *m/z* 1,037.3, eluting at 3.65 min, (**B**) anhydro-44-MG [M–H]- *m/z* 1,019.3, eluting at 3.75 min, and (**C**) dianhydro-44-MG [M–H]- *m/z* 1,001.3, eluting at 3.95 min.

Table S1. Cell quotas of the algal CTX metabolites produced by 34 isolates representing 13 *Gambierdiscus,* five *Coolia* and two *Fukuyoa* species.

Culture ID	Scientific name	P-CTX (pg/cell)				iso-P-CTX (pg/cell)		M-seco-CTX (D/ND)		
		**-3B**	**-3C**	**-4A**	**-4B** [a]	**-3B/C** [b]	**-4A/B** [a]	**-3B/C**	**-3B/C CH_3 acetate**	**-4A/B**
CAWD149	*G. australes*	<0.01	<0.01	<0.01	<0.01	<0.01	<0.01	ND	ND	ND
CAWD381	*G. australes*	<0.01	<0.01	<0.01	<0.01	<0.01	<0.01	ND	ND	ND
CCMP401	*G. belizeanus*	<0.01	<0.01	<0.01	<0.01	<0.01	<0.01	ND	ND	ND
CAWD301	*G. caribaeus*	<0.01	<0.01	<0.01	<0.01	<0.01	<0.01	ND	ND	ND
CAWD237	*G. carpenteri* [c]	<0.01	<0.01	<0.01	<0.01	<0.01	<0.01	ND	ND	ND
CAWD237	*G. carpenteri*	<0.01	<0.01	<0.01	<0.01	<0.01	<0.01	ND	ND	ND
CAWD364	*G. carpenteri*	<0.01	<0.01	<0.01	<0.01	<0.01	<0.01	ND	ND	ND
CAWD232	*G. cheloniae*	<0.01	<0.01	<0.01	<0.01	<0.01	<0.01	ND	ND	ND
CAWD236	*G. cheloniae*	<0.01	<0.01	<0.01	<0.01	<0.01	<0.01	ND	ND	ND
CAWD368	*G. holmesii*	<0.01	<0.01	<0.01	<0.01	<0.01	<0.01	ND	ND	ND
CAWD242	*G. honu*	<0.01	<0.01	<0.01	<0.01	<0.01	<0.01	ND	ND	ND
CAWD250	*G. honu*	<0.01	<0.01	<0.01	<0.01	<0.01	<0.01	ND	ND	ND
NIES-4120	*G. jejuensis*	<0.01	<0.01	<0.01	<0.01	<0.01	<0.01	ND	ND	ND
NIES-4120	*G. jejuensis* [d]	<0.01	<0.01	<0.01	<0.01	<0.01	<0.01	ND	ND	ND
CAWD336	*G. lapillus*	<0.01	<0.01	<0.01	<0.01	<0.01	<0.01	ND	ND	ND
CAWD338	*G. lapillus*	<0.01	<0.01	<0.01	<0.01	<0.01	<0.01	ND	ND	ND
CAWD369	*G. lewisii*	<0.01	<0.01	<0.01	<0.01	<0.01	<0.01	ND	ND	ND
CAWD227	*G. pacificus*	<0.01	<0.01	<0.01	<0.01	<0.01	<0.01	ND	ND	ND
CAWD337	*G. pacificus*	<0.01	<0.01	<0.01	<0.01	<0.01	<0.01	ND	ND	ND
CAWD212	*G. polynesiensis*	**1.1**	**0.19**	**0.13**	**0.04**	**5.1**	**1.3**	ND	ND	ND
CAWD267	*G. polynesiensis*	**0.82**	**0.14**	**0.05**	**0.02**	**7.8**	**2.0**	ND	ND	ND
CAWD429	*G. scabrosus*	<0.01	<0.01	<0.01	<0.01	<0.01	<0.01	ND	ND	ND
CAWD429	*G. scabrosus* [d]	<0.01	<0.01	<0.01	<0.01	<0.01	<0.01	ND	ND	ND
K070922_1	*G. scabrosus*	<0.01	<0.01	<0.01	<0.01	<0.01	<0.01	ND	ND	ND
CAWD385	*C. canariensis*	<0.01	<0.01	<0.01	<0.01	<0.01	<0.01	ND	ND	ND
CAWD387	*C. canariensis*	<0.01	<0.01	<0.01	<0.01	<0.01	<0.01	ND	ND	ND
CAWD154	*C. malayensis*	<0.01	<0.01	<0.01	<0.01	<0.01	<0.01	ND	ND	ND
CAWD175	*C. malayensis*	<0.01	<0.01	<0.01	<0.01	<0.01	<0.01	ND	ND	ND
CAWD60	*C. monotis*	<0.01	<0.01	<0.01	<0.01	<0.01	<0.01	ND	ND	ND
UTS4	*C. palmyrensis*	<0.01	<0.01	<0.01	<0.01	<0.01	<0.01	ND	ND	ND
UTS25	*C. palmyrensis*	<0.01	<0.01	<0.01	<0.01	<0.01	<0.01	ND	ND	ND

CAWD384	*C. tropicalis*	<0.01	<0.01	<0.01	<0.01	<0.01	<0.01	ND	ND	ND
CAWD388	*C. tropicalis*	<0.01	<0.01	<0.01	<0.01	<0.01	<0.01	ND	ND	ND
CAWD238	*F. paulensis*	<0.01	<0.01	<0.01	<0.01	<0.01	<0.01	ND	ND	ND
CAWD306	*F. paulensis*	<0.01	<0.01	<0.01	<0.01	<0.01	<0.01	ND	ND	ND
S044	*F. ruetzeri*	<0.01	<0.01	<0.01	<0.01	<0.01	<0.01	ND	ND	ND
S051	*F. ruetzeri*	<0.01	<0.01	<0.01	<0.01	<0.01	<0.01	ND	ND	ND

G. = Gambierdiscus; C. = Coolia; F. = Fukuyoa; P-CTX = Pacific ciguatoxin; CH_3 = methyl; D = detected; ND = not detected (detection limit unknown as reference material was not available).

All isolates were grown in f/2 media, unless marked with a specific footnote.

[a] Quantified using an LC-MS/MS calibrated reference standard of P-CTX-4A, with a relative response factor of 1.

[b] Quantified using an LC-MS/MS calibrated reference standard of P-CTX-3B, with a relative response factor of 1.

[c] Grown in K media.

[d] Grown in IMK/2 media.

Table S2. Qualitative analysis of C-CTX and I-CTX produced by 34 isolates representing 13 *Gambierdiscus*, five *Coolia* and two *Fukuyoa* species.

Culture ID	Scientific name	D/ND						
		C-CTX-1/2	C-CTX-3/4	C-CTX-5	I-CTX-1/2	I-CTX-3/4	I-CTX-5	I-CTX-6
CAWD149	*G. australes*	ND	ND	ND	ND	ND	ND	ND
CAWD381	*G. australes*	ND	ND	ND	ND	ND	ND	ND
CCMP401	*G. belizeanus*	ND	ND	ND	ND	ND	ND	ND
CAWD301	*G. caribaeus*	ND	ND	ND	ND	ND	ND	ND
CAWD237	*G. carpenteri* [a]	ND	ND	ND	ND	ND	ND	ND
CAWD237	*G. carpenteri*	ND	ND	ND	ND	ND	ND	ND
CAWD364	*G. carpenteri*	ND	ND	ND	ND	ND	ND	ND
CAWD232	*G. cheloniae*	ND	ND	ND	ND	ND	ND	ND
CAWD236	*G. cheloniae*	ND	ND	ND	ND	ND	ND	ND
CAWD368	*G. holmesii*	ND	ND	ND	ND	ND	ND	ND
CAWD242	*G. honu*	ND	ND	ND	ND	ND	ND	ND
CAWD250	*G. honu*	ND	ND	ND	ND	ND	ND	ND
NIES-4120	*G. jejuensis*	ND	ND	ND	ND	ND	ND	ND
NIES-4120	*G. jejuensis* [b]	ND	ND	ND	ND	ND	ND	ND
CAWD336	*G. lapillus*	ND	ND	ND	ND	ND	ND	ND
CAWD338	*G. lapillus*	ND	ND	ND	ND	ND	ND	ND
CAWD369	*G. lewisii*	ND	ND	ND	ND	ND	ND	ND
CAWD227	*G. pacificus*	ND	ND	ND	ND	ND	ND	ND
CAWD337	*G. pacificus*	ND	ND	ND	ND	ND	ND	ND
CAWD212	*G. polynesiensis*	ND	ND	ND	ND	ND	ND	ND
CAWD267	*G. polynesiensis*	ND	ND	ND	ND	ND	ND	ND
CAWD429	*G. scabrosus*	ND	ND	ND	ND	ND	ND	ND
CAWD429	*G. scabrosus* [b]	ND	ND	ND	ND	ND	ND	ND
K070922_1	*G. scabrosus*	ND	ND	ND	ND	ND	ND	ND
CAWD385	*C. canariensis*	ND	ND	ND	ND	ND	ND	ND
CAWD387	*C. canariensis*	ND	ND	ND	ND	ND	ND	ND
CAWD154	*C. malayensis*	ND	ND	ND	ND	ND	ND	ND
CAWD175	*C. malayensis*	ND	ND	ND	ND	ND	ND	ND
CAWD60	*C. monotis*	ND	ND	ND	ND	ND	ND	ND
UTS4	*C. palmyrensis*	ND	ND	ND	ND	ND	ND	ND
UTS25	*C. palmyrensis*	ND	ND	ND	ND	ND	ND	ND
CAWD384	*C. tropicalis*	ND	ND	ND	ND	ND	ND	ND

CAWD388	*C. tropicalis*	ND	ND	ND	ND	ND	ND	ND
CAWD238	*F. paulensis*	ND	ND	ND	ND	ND	ND	ND
CAWD306	*F. paulensis*	ND	ND	ND	ND	ND	ND	ND
S044	*F. ruetzeri*	ND	ND	ND	ND	ND	ND	ND
S051	*F. ruetzeri*	ND	ND	ND	ND	ND	ND	ND

G. = Gambierdiscus; C. = Coolia; F. = Fukuyoa; C-CTX = Caribbean ciguatoxin; I-CTX = Indian ciguatoxin; D= detected; ND = not detected (detection limit unknown as reference material was not available).

All isolates were grown in f/2 media, unless marked with a specific footnote.

[a] Grown in K media.

[b] Grown in IMK/'2 media.

Table S3. Cell quotas of maitotoxins produced by 34 isolates representing 13 *Gambierdiscus*, five *Coolia* and two *Fukuyoa* species.

Culture ID	Scientific name	pg/cell					
		**MTX-1**	**MTX-2**	**MTX-4**	**MTX-5** [a]	**MTX-6** [a]	**MTX-7** [a]
CAWD149	*G. australes*	**6**	<0.01	<0.01	**0.2**	<0.01	<0.01
CAWD381	*G. australes*	**9**	<0.01	<0.01	**0.1**	<0.01	<0.01
CCMP401	*G. belizeanus*	<0.01	<0.01	<0.01	<0.01	<0.01	<0.01
CAWD301	*G. caribaeus*	<0.01	<0.01	<0.01	<0.01	<0.01	<0.01
CAWD237	*G. carpenteri* [b]	<0.01	<0.01	<0.01	<0.01	<0.01	<0.01
CAWD237	*G. carpenteri*	<0.01	<0.01	<0.01	<0.01	<0.01	<0.01
CAWD364	*G. carpenteri*	<0.01	<0.01	<0.01	<0.01	<0.01	<0.01
CAWD232	*G. cheloniae*	<0.01	<0.01	<0.01	<0.01	**4**	<0.01
CAWD236	*G. cheloniae*	<0.01	<0.01	<0.01	<0.01	**5**	<0.01
CAWD368	*G. holmesii*	<0.01	<0.01	<0.01	<0.01	<0.01	<0.01
CAWD242	*G. honu*	<0.01	<0.01	<0.01	<0.01	<0.01	**14**
CAWD250	*G. honu*	<0.01	<0.01	<0.01	<0.01	<0.01	**2**
NIES-4120	*G. jejuensis*	<0.01	<0.01	<0.01	<0.01	<0.01	<0.01
NIES-4120	*G. jejuensis* [c]	<0.01	<0.01	<0.01	<0.01	<0.01	<0.01
CAWD336	*G. lapillus*	<0.01	<0.01	<0.01	<0.01	<0.01	<0.01
CAWD338	*G. lapillus*	<0.01	<0.01	<0.01	<0.01	<0.01	<0.01
CAWD369	*G. lewisii*	<0.01	<0.01	<0.01	<0.01	<0.01	<0.01
CAWD227	*G. pacificus*	<0.01	<0.01	<0.01	<0.01	<0.01	<0.01
CAWD337	*G. pacificus*	<0.01	<0.01	<0.01	<0.01	<0.01	<0.01
CAWD212	*G. polynesiensis*	<0.01	<0.01	<0.01	<0.01	<0.01	<0.01
CAWD267	*G. polynesiensis*	<0.01	<0.01	<0.01	<0.01	<0.01	<0.01
CAWD429	*G. scabrosus*	<0.01	<0.01	<0.01	<0.01	<0.01	<0.01
CAWD429	*G. scabrosus* [c]	<0.01	<0.01	<0.01	<0.01	<0.01	<0.01
K070922_1	*G. scabrosus*	<0.01	<0.01	<0.01	<0.01	<0.01	<0.01
CAWD385	*C. canariensis*	<0.01	<0.01	<0.01	<0.01	<0.01	<0.01
CAWD387	*C. canariensis*	<0.01	<0.01	<0.01	<0.01	<0.01	<0.01
CAWD154	*C. malayensis*	<0.01	<0.01	<0.01	<0.01	<0.01	<0.01
CAWD175	*C. malayensis*	<0.01	<0.01	<0.01	<0.01	<0.01	<0.01
CAWD60	*C. monotis*	<0.01	<0.01	<0.01	<0.01	<0.01	<0.01
UTS4	*C. palmyrensis*	<0.01	<0.01	<0.01	<0.01	<0.01	<0.01
UTS25	*C. palmyrensis*	<0.01	<0.01	<0.01	<0.01	<0.01	<0.01
CAWD384	*C. tropicalis*	<0.01	<0.01	<0.01	<0.01	<0.01	<0.01

CAWD388	*C. tropicalis*	<0.01	<0.01	<0.01	<0.01	<0.01	<0.01
CAWD238	*F. paulensis*	<0.01	<0.01	<0.01	<0.01	<0.01	<0.01
CAWD306	*F. paulensis*	<0.01	<0.01	<0.01	<0.01	<0.01	<0.01
S044	*F. ruetzeri*	<0.01	<0.01	<0.01	<0.01	<0.01	<0.01
S051	*F. ruetzeri*	<0.01	<0.01	<0.01	<0.01	<0.01	<0.01

G. = *Gambierdiscus*; *C.* = *Coolia*; *F.* = *Fukuyoa;* MTX = Maitotoxin.
All isolates were grown in f/2 media, unless marked with a specific footnote.
[a] Quantified using MTX-1 reference material, with a relative response factor of 1.
[b] Grown in K media.
[c] Grown in IMK/2 media.

Table S4. Cell quotas and qualitative analysis of gambierones produced by 34 isolates representing 13 *Gambierdiscus*, five *Coolia* and two *Fukuyoa* species.

Culture ID	Scientific name	pg/cell	D/ND				pg/cell	D/ND				
		G	Anhydro G	Dianhydro G	sulfo-G	DiH-sulfo-G	44-MG	12,13-diH-44-MG	29-MG	38-deOH-44-MG	38-deOH-12,13-diH-44-MG	Dianhydro 44-MG
CAWD149	*G. australes*	<0.01 [a]	ND	ND	ND	ND	**259** [a]	**D**	ND	**D**	ND	ND
CAWD381	*G. australes*	<0.01	ND	ND	ND	ND	**160**	**D**	ND	**D**	ND	ND
CCMP401	*G. belizeanus*	**540**	**D**	ND	ND	ND	**53**	**D**	ND	ND	**D**	ND
CAWD301	*G. caribaeus*	<0.01 [a]	ND	ND	ND	ND	**44** [a]	**D**	**D**	**D**	ND	ND
CAWD237	*G. carpenter* [b]	**87** [a]	**D**	ND	ND	ND	**74** [a]	**D**	ND	**D**	**D**	ND
CAWD237	*G. carpenteri*	**65** [a]	**D**	ND	ND	ND	**45** [a]	**D**	ND	**D**	**D**	ND
CAWD364	*G. carpenteri*	<0.01	ND	ND	ND	ND	**441**	**D**	ND	ND	**D**	ND
CAWD232	*G. cheloniae*	**55** [a]	**D**	ND	ND	ND	**26** [a]	**D**	ND	ND	**D**	ND
CAWD236	*G. cheloniae*	**358**	**D**	ND	ND	ND	**43**	**D**	ND	ND	**D**	ND
CAWD368	*G. holmesii*	**20** [a]	**D**	ND	ND	ND	**97** [a]	**D**	ND	ND	**D**	ND
CAWD242	*G. honu*	**38** [a]	**D**	ND	ND	ND	**182** [a]	**D**	ND	**D**	**D**	ND
CAWD250	*G. honu*	**42**	**D**	ND	ND	ND	**54**	**D**	**D**	**D**	**D**	ND
NIES-4120	*G. jejuensis*	<0.01	ND	ND	ND	ND	**71**	**D**	**D**	**D**	**D**	ND
NIES-4120	*G. jejuensis* [c]	<0.01	ND	ND	ND	ND	**72**	**D**	**D**	**D**	**D**	ND
CAWD336	*G. lapillus*	<0.01 [a]	ND	ND	ND	**D**	**46** [a]	**D**	**D**	**D**	**D**	ND
CAWD338	*G. lapillus*	<0.01 [a]	ND	ND	ND	**D**	**270** [a]	**D**	**D**	**D**	**D**	ND
CAWD369	*G. lewisii*	**1** [a]	ND	ND	ND	ND	**68** [a]	**D**	ND	**D**	**D**	ND
CAWD227	*G. pacificus*	**8**	**D**	ND	ND	ND	**65**	**D**	**D**	**D**	**D**	ND
CAWD337	*G. pacificus*	**1** [a]	**D**	ND	ND	ND	**100** [a]	**D**	**D**	**D**	**D**	ND
CAWD212	*G. polynesiensis*	**13** [a]	**D**	ND	ND	ND	**29** [a]	**D**	**D**	ND	**D**	ND
CAWD267	*G. polynesiensis*	**13** [a]	**D**	ND	ND	ND	**44** [a]	**D**	**D**	ND	**D**	ND
CAWD429	*G. scabrosus*	**131**	**D**	ND	ND	ND	**17**	**D**	**D**	ND	**D**	ND
CAWD429	*G. scabrosus* [c]	**102**	**D**	ND	ND	ND	**17**	**D**	**D**	ND	**D**	ND
K070922_1	*G. scabrosus*	**166**	**D**	ND	ND	ND	**32**	**D**	**D**	ND	**D**	ND
CAWD385	*C. canariensis*	<0.01	ND	ND	ND	ND	<0.01	ND	ND	ND	ND	ND
CAWD387	*C. canariensis*	<0.01	ND	ND	ND	ND	<0.01	ND	ND	ND	ND	ND
CAWD154	*C. malayensis*	**2** [a]	**D**	ND	ND	ND	**9** [a]	**D**	**D**	ND	**D**	ND
CAWD175	*C. malayensis*	**17** [a]	**D**	ND	ND	ND	**24** [a]	**D**	**D**	ND	**D**	ND
CAWD60	*C. monotis*	<0.01	ND	ND	ND	ND	<0.01	ND	ND	ND	ND	ND
UTS4	*C. palmyrensis*	<0.01	ND	ND	ND	ND	<0.01	ND	ND	ND	ND	ND
UTS25	*C. palmyrensis*	<0.01	ND	ND	ND	ND	<0.01	ND	ND	ND	ND	ND

CAWD384	*C. tropicalis*	<0.01 [a]	ND	ND	ND	ND	**14** [a]	**D**	**D**	ND	**D**	ND
CAWD388	*C. tropicalis*	<0.01 [a]	ND	ND	ND	ND	**15** [a]	**D**	**D**	ND	**D**	ND
CAWD238	*F. paulensis*	<0.01 [a]	ND	ND	ND	ND	**5** [a]	**D**	**D**	ND	**D**	ND
CAWD306	*F. paulensis*	<0.01 [a]	ND	ND	ND	ND	**65** [a]	**D**	**D**	ND	**D**	ND
S044	*F. ruetzeri*	**8** [a]	**D**	ND	ND	ND	**12** [a]	**D**	ND	ND	**D**	ND
S051	*F. ruetzeri*	**6** [a]	**D**	ND	ND	ND	**13** [a]	**D**	ND	ND	**D**	ND

G. = *Gambierdiscus; C.* = *Coolia; F.* = *Fukuyoa;* 44-MG = 44-Methylgambierone; G = gambierone; DiH = dihydro; deOH = deoxy; D = detected; ND = not detected (detection limit unknown as reference material was not available).

All isolates were grown in f/2 media, unless marked with a specific footnote.

[a] Results transcribed from Murray *et al*., 2021.

[b] Grown in K media.

[c] Grown in IMK/2 media.

Table S5. Qualitative analysis of six additional metabolites produced by 34 isolates representing 13 *Gambierdiscus*, five *Coolia* and two *Fukuyoa* species.

Culture ID	Scientific name	D/ND					
		**Gambieroxide** [a]	**Gambierol** [a]	**Gambieric acid A** [a]	**Gambieric acid B** [a]	**Gambieric acid C** [a]	**Gambieric acid D** [a]
CAWD149	*G. australes*	ND	ND	**D**	**D**	ND	ND
CAWD381	*G. australes*	ND	ND	**D**	**D**	**D**	**D**
CCMP401	*G. belizeanus*	ND	ND	**D**	**D**	ND	ND
CAWD301	*G. caribaeus*	ND	ND	**D**	ND	ND	ND
CAWD237	*G. carpenteri* [b]	ND	ND	**D**	ND	ND	ND
CAWD237	*G. carpenteri*	ND	ND	**D**	ND	ND	ND
CAWD364	*G. carpenteri*	ND	ND	**D**	ND	ND	ND
CAWD232	*G. cheloniae*	ND	ND	**D**	ND	ND	ND
CAWD236	*G. cheloniae*	ND	ND	ND	ND	ND	ND
CAWD368	*G. holmesii*	ND	ND	**D**	ND	ND	ND
CAWD242	*G. honu*	ND	ND	ND	ND	ND	ND
CAWD250	*G. honu*	ND	ND	ND	**D**	ND	ND
NIES-4120	*G. jejuensis*	ND	ND	ND	**D**	ND	ND
NIES-4120	*G. jejuensis* [c]	ND	ND	ND	**D**	ND	ND
CAWD336	*G. lapillus*	ND	ND	**D**	ND	ND	ND
CAWD338	*G. lapillus*	ND	ND	**D**	ND	ND	ND
CAWD369	*G. lewisii*	**D**	ND	**D**	ND	ND	ND
CAWD227	*G. pacificus*	**D**	ND	**D**	ND	ND	ND
CAWD337	*G. pacificus*	**D**	ND	**D**	ND	ND	ND
CAWD212	*G. polynesiensis*	ND	ND	**D**	ND	ND	ND
CAWD267	*G. polynesiensis*	ND	ND	**D**	ND	ND	ND
CAWD429	*G. scabrosus*	ND	ND	ND	ND	ND	ND
CAWD429	*G. scabrosus* [c]	ND	ND	ND	ND	ND	ND
K070922_1	*G. scabrosus*	ND	ND	ND	ND	ND	ND
CAWD385	*C. canariensis*	ND	ND	ND	ND	ND	ND
CAWD387	*C. canariensis*	ND	ND	ND	ND	ND	ND
CAWD154	*C. malayensis*	ND	ND	ND	ND	ND	ND
CAWD175	*C. malayensis*	ND	ND	ND	ND	ND	ND
CAWD60	*C. monotis*	ND	ND	ND	ND	ND	ND
UTS4	*C. palmyrensis*	ND	ND	ND	ND	ND	ND
UTS25	*C. palmyrensis*	ND	ND	ND	ND	ND	ND
CAWD384	*C. tropicalis*	ND	ND	ND	ND	ND	ND

CAWD388	*C. tropicalis*	ND	ND	ND	ND	ND	ND
CAWD238	*F. paulensis*	ND	ND	ND	ND	ND	ND
CAWD306	*F. paulensis*	ND	ND	ND	ND	ND	ND
S044	*F. ruetzeri*	ND	ND	ND	ND	ND	ND
S051	*F. ruetzeri*	ND	ND	ND	ND	ND	ND

G. = *Gambierdiscus*; *C.* = *Coolia*; *F.* = *Fukuyoa*; D = detected; ND = Not detected (detection limit unknown as reference material was not available).
All isolates were grown in f/2 media, unless marked with a specific footnote.
[a] Analysis was performed using published MRM transitions for these compounds and detections are tentative only until reference material is available.
[b] Grown in K media.
[c] Grown in IMK/2 media.

Table S6. Metabolite spike recoveries in representative isolates of *Gambierdiscus*, *Coolia* and *Fukuyoa*.

Analogue	Spike level (ng/mL)	Recovery (%)		
		G. caribaeus **CAWD301**	***C. malayensis*** **CAWD154**	***F. paulensis*** **CAWD238**
P-CTX-3B	10	96	109	112
	2	91	102	106
P-CTX-3C	10	92	101	101
	2	91	94	94
P-CTX-4A	10	91	98	102
	2	91	93	97
P-CTX-1B	10	93	106	105
	2	92	104	93
MTX-1	50	98	98	113
	20	105	100	78
MTX-6	50	96	104	108
	20	87	91	99
MTX-7	50	121	136	136
	20	92	124	118
Gambierone	500	116	112	124
	100	118	122	131
44-MG	500	63	53	63
	100	57	53	66

G. = *Gambierdiscus; C.* = *Coolia; F.* = *Fukuyoa;* P-CTX = Pacific ciguatoxin; MTX = maitotoxin; 44-MG = 44-methylgambierone.

Table S7. Summary of the toxicity information (*in vivo* and *in vitro*) available for the ciguatoxins and maitotoxins.

Toxin class	Metabolite	Toxicity	
		Mouse (LD_{50}; i.p injection; µg/kg)	N2a-CBA (EC_{50})
Pacific Ciguatoxins (P-CTXs) Type I	P-CTX-4A	1.4 [1]	12.4 pg/mL [2]
	P-CTX-4B	3.6 [1]	23.3 pg/mL [1]
	iso-P-CTX-4A/B	–	–
	P-CTX-1B	0.36 [1]	1.08 pg/mL [2]
	52-epi-54-deoxy-CTX-1B	0.7 [1]	2.98 pg/mL [2]
	54-deoxy-CTX-1B	1.6 [3]	2.73 pg/mL [2]
	7-oxo-CTX-1B	–	–
	7-hydroxy-CTX-1B	–	–
	4-hydroxy-7-oxo-CTX-1B	–	–
Pacific Ciguatoxins (P-CTXs) Type II	P-CTX-3B	–	–
	P-CTX-3C	1.2 [1]	2.03 pg/mL [2]
	iso-P-CTX-3B/C	–	–
	51-hydroxy-3C	0.20 [1]	1.18 pg/mL [2]
	M-seco-CTX-3B	–	–
	M-seco-CTX-3C	–	–
	M-seco-CTX-3C methyl acetal	–	–
	51-hydroxy-3-oxo-CTX-3C	–	–
	A-seco-51-hydroxy-CTX-3C	–	–
	2,3-dihydroxy-CTX-3B	–	–
	2,3-dihydroxy-CTX-3C	–	–
	2,3,51-trihydroxy-CTX-3C	–	–
	2-hydroxy-CTX-3C	–	–
Caribbean ciguatoxins (C-CTXs)	C-CTX-1	3.6 [4]	–
	C-CTX-2	~1 [4]	–
	C-CTX-3	–	–
	C-CTX-4	–	–
	C-CTX-5	–	–
Indian ciguatoxins (I-CTXs)	I-CTX-1	–	–
	I-CTX-2	–	–
	I-CTX-3	–	–
	I-CTX-4	–	–
	I-CTX-6	–	–
	I-CTX-1	–	–
Maitotoxins (MTXs)	MTX-1	0.05 [5]	6 ng/mL [6]
	MTX-2	0.08 [7]	–
	MTX-4	–	–
	MTX-5	–	–
	MTX-6	6.45 [8] [a]	–
	MTX-7	0.235 [8]	–

MBA = Mouse bioassay; N2a CBA = neuroblastoma cell-based assay; i.p. = intraperitoneal injection
[a] This compound was semi-pure, therefore, this result will be much lower.

Table S8. Summary of the toxicity information (*in vivo* and *in vitro*) available for the gambierones, gambieroxide, gambierol and the gambieric acids.

Toxin class	Metabolite	Toxicity	
		Mouse (LD_{50}; i.p. injection; μg/kg)	N2a-CBA (EC_{50})
Gambierones	Gambierone	2,400 [9]	1.12 μg/mL [10]
	Anhydrogambierone	–	–
	Dianhydrogambierone	–	–
	Sulfo-gambierone	–	–
	Dihydro-sulfo-gambierone	–	–
	44-MG	20,000–38,000 [9]	>4.76 μg/mL [10]
	29-MG	–	–
	12,13-dihydro-44-MG	–	–
	38-deoxy-44-MG	–	–
	38-deoxy-12,13-dihydro-44-MG	–	–
	Dianhydro-44-MG	–	–
–	Gambieroxide	–	–
–	Gambierol	MLD 50 [11]	–
Gambieric acids	Gambieric acid A	MLD >1,000 [12]	–
	Gambieric acid B	MLD >1,000 [12]	–
	Gambieric acid C	–	–
	Gambieric acid D	–	–

MBA = Mouse bioassay; N2a CBA = neuroblastoma cell-based assay; 44-MG = 44-methylgambierone; MLD = minimum lethal dose; i.p. = intraperitoneal injection.

Table S9. List of the MRM transitions and CEs used for the Type I Pacific ciguatoxins monitored.

Analogue	Chemical Formula	mw (Da) [a]	Precursor ion	MRM transition	CE (eV)
P-CTX-4A/B and isomer	$C_{60}H_{84}O_{16}$	1,060.6	$[M+H]^+$	1,061.6>125.1<br>1,061.6>155.1	50
M-seco-CTX-4A/B	$C_{60}H_{86}O_{17}$	1,078.6	$[M+H]^+$	1,079.6>143.1<br>1,079.6>173.1	50
P-CTX-1B	$C_{60}H_{86}O_{19}$	1,110.6	$[M+NH_4]^+$	1,128.6>95.1	65
				1,128.6>109	55
			$[M+Na]^+$	1,133.6>1,133.6	55
52-epi-54-deoxy-CTX-1B	$C_{60}H_{86}O_{18}$	1,094.6	$[M+NH_4]^+$	1,112.6>1,041.8	25
				1,112.6>1,077.8	20
			$[M+Na]^+$	1,117.6>1,117.6	55
54-deoxy-CTX-1B	$C_{60}H_{86}O_{18}$	1,094.6	$[M+NH_4]^+$	1,112.6>1,041.8	25
				1,112.6>1,077.8	20
			$[M+Na]^+$	1,117.6>1,117.6	55
7-oxo-CTX-1B	$C_{60}H_{86}O_{20}$	1,126.6	$[M+NH_4]^+$	1,144.6>95.1	65
				1,144.6>109	50
			$[M+Na]^+$	1,149.6>1,149.6	50
7-hydroxy-CTX-1B	$C_{60}H_{88}O_{20}$	1,128.6	$[M+NH_4]^+$	1,146.6>95.1	65
				1,146.6>109	50
			$[M+Na]^+$	1,151.6>1,151.6	50
4-hydroxy-7-oxo-CTX-1B	$C_{60}H_{88}O_{21}$	1,144.6	$[M+NH_4]^+$	1,162.6>95.1	65
				1,162.6>109	50
			$[M+Na]^+$	1,167.6>1,167.6	50

mw = Molecular weight; MRM = multiple reaction monitoring; CE = collision energy; P-CTX = Pacific ciguatoxin.
[a] Reported as the monoisotopic mass.

Table S10. List of the MRM transitions and CEs used for the Type II Pacific ciguatoxins monitored.

Analogue	Chemical Formula	mw (Da) [a]	Precursor ion	MRM transition	CE (eV)
P-CTX-3B/C and isomer	$C_{57}H_{82}O_{16}$	1,022.6	$[M+H]^+$	1,023.6>125.1<br>1,023.6>155.1	50
M-seco-CTX-3B/C	$C_{57}H_{84}O_{17}$	1,040.6	$[M+H]^+$	1,041.6>143.1<br>1,041.6>173.1	50
M-seco-CTX-3C methyl acetate	$C_{58}H_{86}O_{17}$	1,054.6	$[M+H]^+$	1,055.6>157.1<br>1,055.6>187.1	50
51-hydroxy-CTX-3C	$C_{57}H_{82}O_{17}$	1,038.6	$[M+H]^+$	1,039.6>141.1<br>1,039.6>171.1	50
			$[M+Na]^+$	1,061.6>1,061.6	50
2-hydroxy-CTX-3C	$C_{57}H_{84}O_{17}$	1,040.6	$[M+H]^+$	1,039.6>125.1<br>1,039.5>155.1	50
			$[M+Na]^+$	1,061.6>1,061.6	50
51-hydroxy-2-oxo-CTX-3C	$C_{57}H_{82}O_{18}$	1,054.6	$[M+H]^+$	1,055.6>141.1<br>1,055.6>171.1	50
			$[M+Na]^+$	1,077.6>1,077.6	50
2,3-dihydroxy-CTX-3B/C	$C_{57}H_{84}O_{18}$	1,056.6	$[M+H]^+$	1,057.6>125.1<br>1,057.6>155.1	50
			$[M+Na]^+$	1,079.6>1,079.6	50
2,3,51-trihydroxy-CTX-3C	$C_{57}H_{84}O_{19}$	1,072.6	$[M+H]^+$	1,073.6>141.1<br>1,073.6>171.1	50
			$[M+Na]^+$	1,095.6>1,095.6	50
A-seco-51-hydroxy-CTX-3C	$C_{57}H_{86}O_{18}$	1,092.6	$[M+H]^+$	1,093.6>141.1<br>1,093.6>171.1	50
			$[M+Na]^+$	1,115.6>1,115.6	50

mw = Molecular weight; MRM = multiple reaction monitoring; CE = collision energy; CTX = Ciguatoxin.
[a] Reported as the monoisotopic mass.

Table S11. List of the MRM transitions and CEs used for the Caribbean and Indian ciguatoxins monitored.

Analogue	Chemical Formula	mw (Da) [a]	Precursor ion	MRM transition	CE (eV)
C-CTX-1/2	$C_{62}H_{92}O_{19}$	1,140.6	$[M+NH_4]^+$	1,158.6>1,123.6<br>1,158.6>1,105.6	15
			$[M+H–H_2O]^+$	1,123.6>253.1	50
			$[M+Na]^+$	1,163.6>1,163.6	50
C-CTX-3/4	$C_{62}H_{94}O_{19}$	1,142.6	$[M+NH_4]^+$	1,160.6>1,125.6<br>1,160.6>1,107.6	15
			$[M+H–H_2O]^+$	1,125.6>255.1	50
			$[M+Na]^+$	1,165.6>1,165.6	50
C-CTX-5	$C_{62}H_{90}O_{19}$	1,138.6	$[M+NH_4]^+$	1,156.6>1,121.6<br>1,156.6>1,103.6	15
			$[M+H–H_2O]^+$	1,121.6>253.1	50
			$[M+Na]^+$	1,161.6>1,161.6	50
I-CTX-1/2	$C_{62}H_{92}O_{19}$	1,140.6	$[M+H]^+$	1,141.6>1,123.6	15
			$[M+NH_4]^+$	1,158.6>1,123.6	15
			$[M+Na]^+$	1,163.6>1,163.6	50
I-CTX-3/4	$C_{62}H_{92}O_{20}$	1,156.6	$[M+H]^+$	1,157.6>1,139.6	15
			$[M+NH_4]^+$	1,174.6>1,139.6	15
			$[M+Na]^+$	1,179.6>1,179.6	50
I-CTX-5	$C_{62}H_{90}O_{19}$	1,138.6	$[M+H]^+$	1,139.6>1,121.6	15
			$[M+NH_4]^+$	1,156.6>1,121.6	15
			$[M+Na]^+$	1,161.6>1,161.6	50
I-CTX-6	$C_{62}H_{90}O_{20}$	1,154.6	$[M+H]^+$	1,155.6>1,137.6	15
			$[M+NH_4]^+$	1,172.6>1,137.6	15
			$[M+Na]^+$	1,177.6>1,177.6	50

mw = Molecular weight; MRM = multiple reaction monitoring; CE = collision energy; C-CTX = Caribbean ciguatoxin; I-CTX = Indian ciguatoxin.
[a] Reported as the monoisotopic mass.

Table S12. List of the MRM transitions and CEs used for the maitotoxins monitored.

Analogue	Chemical Formula	mw (Da) [a]	Precursor ion	MRM transition	CE (eV)
MTX-1	$C_{164}H_{256}O_{68}S_2$	3,379.6 [b]	$[M–2H]^{2-}$	1,689.4>1,689.4	80
			$[M–3H]^{3-}$	1,126.1>96.8	100
MTX-2	–	3,298 [b]	$[M–2H]^{2-}$	1,637.5>1,637.5	60
			$[M–3H+Na]^{2-}$	1,648.2>1,648.2	60
			$[M–4H+Na]^{3-}$	1,098.6>96.8	100
MTX-4	$C_{157}H_{241}NO_{68}S_2$	3,292.5 [b]	$[M–2H]^{2-}$	1,645.2>1,645.2	60
			$[M–3H]^{3-}$	1,096.5>96.8	100
MTX-5	$C_{161}H_{252}O_{68}S_2$	3,337.6 [b]	$[M–2H]^{2-}$	1,668.8>>1,668.8	40
			$[M–2H]^{2-}$	1,668.8>96.8	100
MTX-6	$C_{164}H_{256}O_{66}S$	3,313.6 [b]	$[M–2H]^{2-}$	1,656.3>1,656.3	60
			$[M–3H]^{3-}$	1,104.1>96.8	100
MTX-7	$C_{165}H_{258}O_{67}S$	3,343.7 [b]	$[M–2H]^{2-}$	1,671.4>1,671.4	60
			$[M–3H]^{3-}$	1,114.1>96.8	100

mw = Molecular weight; MRM = multiple reaction monitoring; CE = collision energy; MTX = maitotoxin.
[a] Reported as the monoisotopic mass.
[b] Free acid form.

Table S13. List of the MRM transitions and CEs used for the gambierones monitored.

Analogue	Chemical Formula	mw (Da) [a]	Precursor ion	MRM transition	CE (eV)
Gambierone	$C_{51}H_{76}O_{19}S$	1,024.5	$[M–H]^-$	1,023.3>96.8	50
			C-38 fragment	899.2>96.8	50
Anhydrogambierone	$C_{51}H_{74}O_{18}S$	1,006.5	$[M–H]^-$	1,005.3>96.8	50
			C-38 fragment	881.3>96.8	50
Dianhydrogambierone	$C_{51}H_{72}O_{17}S$	988.5	$[M–H]^-$	987.3>96.8	50
			C-38 fragment	863.2>96.8	50
Sulfo-gambierone	$C_{51}H_{76}O_{22}S_2$	1,104.5	$[M–H]^-$	1,103.5>96.8	50
Dihydrosulfo-gambierone	$C_{51}H_{78}O_{22}S_2$	1,106.5	$[M–H]^-$	1,105.5>96.8	50
44- and 29-MG	$C_{52}H_{78}O_{19}S$	1,038.3	$[M–H]^-$	1,037.3>96.8	70
			C-38 fragment	899.2>96.8	50
38-deoxy-44-MG	$C_{52}H_{76}O_{18}S$	1,020.3	$[M–H]^-$	1,019.3>96.8	70
			C-38 fragment	881.3>96.8	50
Dianhydro-44-MG	$C_{52}H_{74}O_{17}S$	1,002.3	$[M–H]^-$	1,001.3>96.8	70
			C-38 fragment	863.2>96.8	50
12,13-dihydro-44-MG	$C_{52}H_{80}O_{19}S$	1,040.5	$[M–H]^-$	1,039.5>96.8	70
38-deoxy-12,13-dihydro-44-MG	$C_{52}H_{78}O_{18}S$	1,022.5	$[M–H]^-$	1,021.5>96.8	70

mw = Molecular weight; MRM = multiple reaction monitoring; CE = collision energy; 44-MG = 44-methylgambierone.
[a] Reported as the monoisotopic mass.

Table S14. List of the MRM transitions and CEs used for the gambieroxide, gambierol and the gambieric acids.

Analogue	Chemical Formula	mw (Da) [a]	Precursor ion	MRM transition	CE (eV)
Gambieroxide	$C_{60}H_{90}O_{22}S$	1,194.6	$[M–H]^-$	1,193.6>96.8	60
Gambierol	$C_{43}H_{64}O_{11}$	756.5	$[M–H]^-$	755.5>755.5	20
			$[M+H]^+$	757.5>757.5	5
			$[M+Na]^+$	779.5>779.5	10
Gambieric acid A	$C_{59}H_{92}O_{16}$	1,056.6	$[M–H]^-$	1,055.6>1,055.6	20
			$[M+H]^+$	1,057.6>1,057.6	5
Gambieric acid B	$C_{60}H_{94}O_{16}$	1,070.7	$[M–H]^-$	1,069.7>1,069.7	20
			$[M+H]^+$	1,071.7>1,071.7	5
Gambieric acid C	$C_{65}H_{100}O_{19}$	1,184.7	$[M–H]^-$	1,183.7>1,183.7	20
			$[M+H]^+$	1,185.7>135.1	49
				1,185.7>1,039.6	17
Gambieric acid D	$C_{66}H_{102}O_{19}$	1,198.7	$[M–H]^-$	1,197.7>1,197.7	20
			$[M+H]^+$	1,199.7>135.1	49
				1,199.7>1,053.6	17

mw = Molecular weight; MRM = multiple reaction monitoring; CE = collision energy.
[a] Reported as the monoisotopic mass.

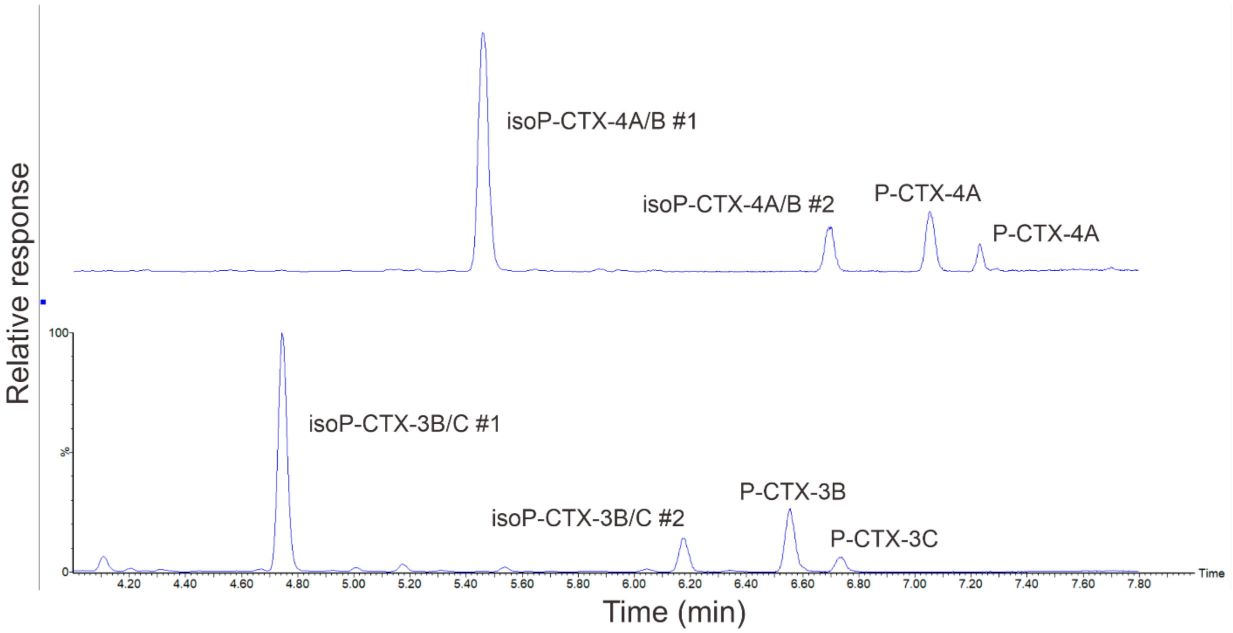

Figure S3. Example chromatogram showing the elution order of the primary P-CTX algal metabolites.

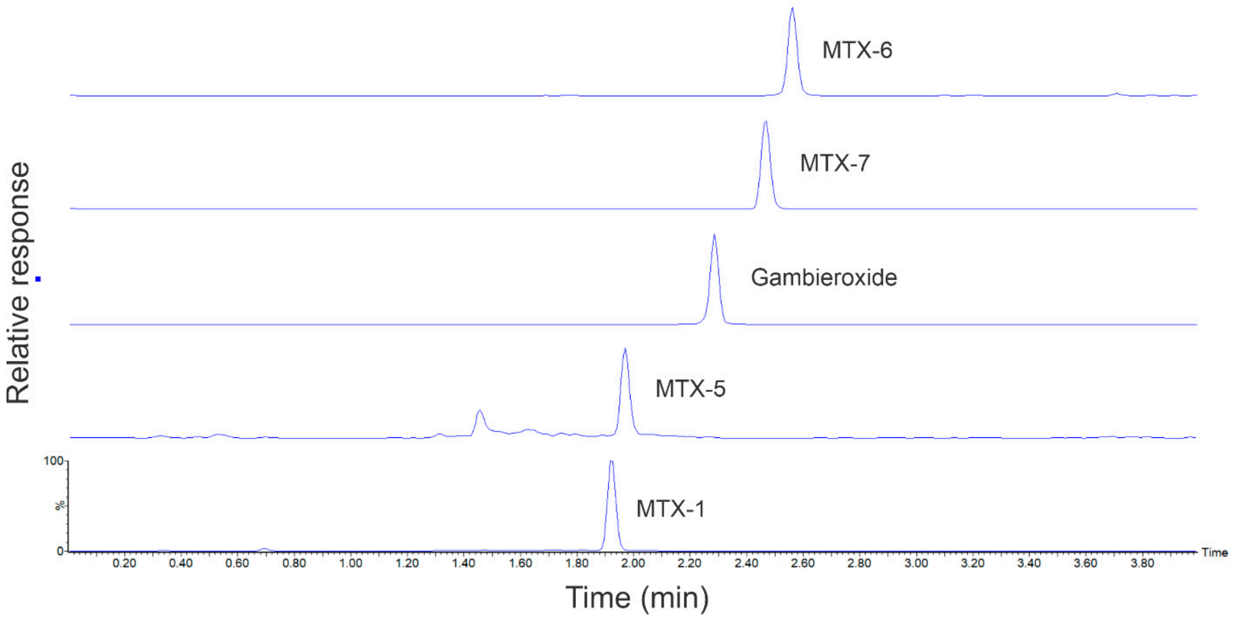

Figure S4. Example chromatogram showing the elution order of the MTXs and gambieroxide metabolites.

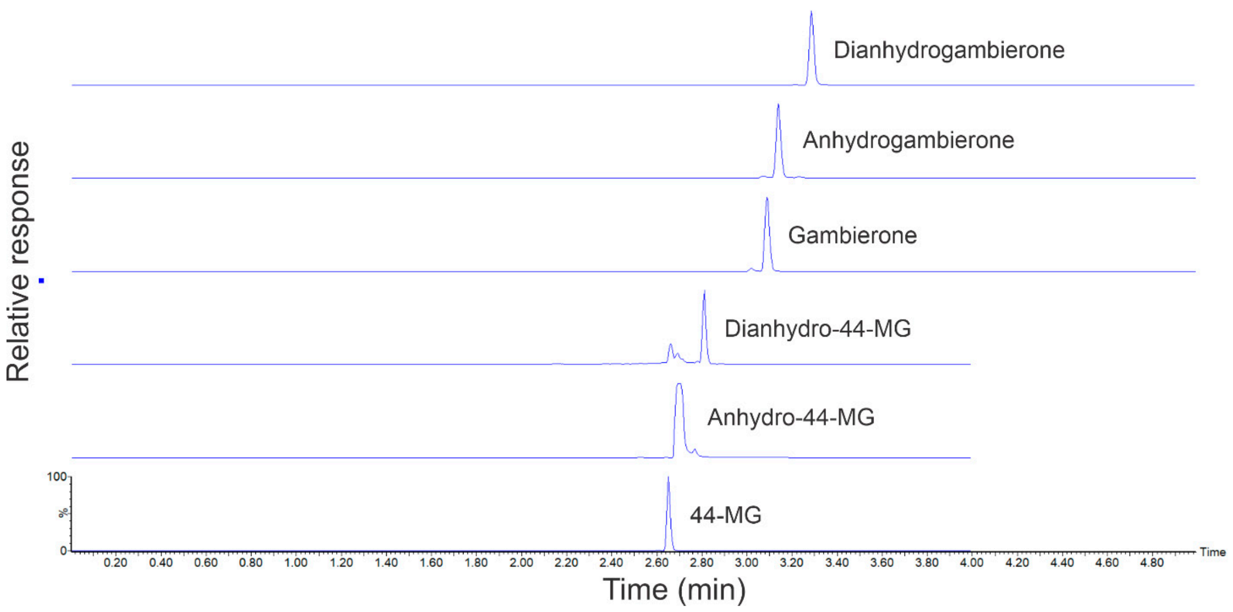

Figure S5. Example chromatogram showing the elution order of the gambierone metabolites.

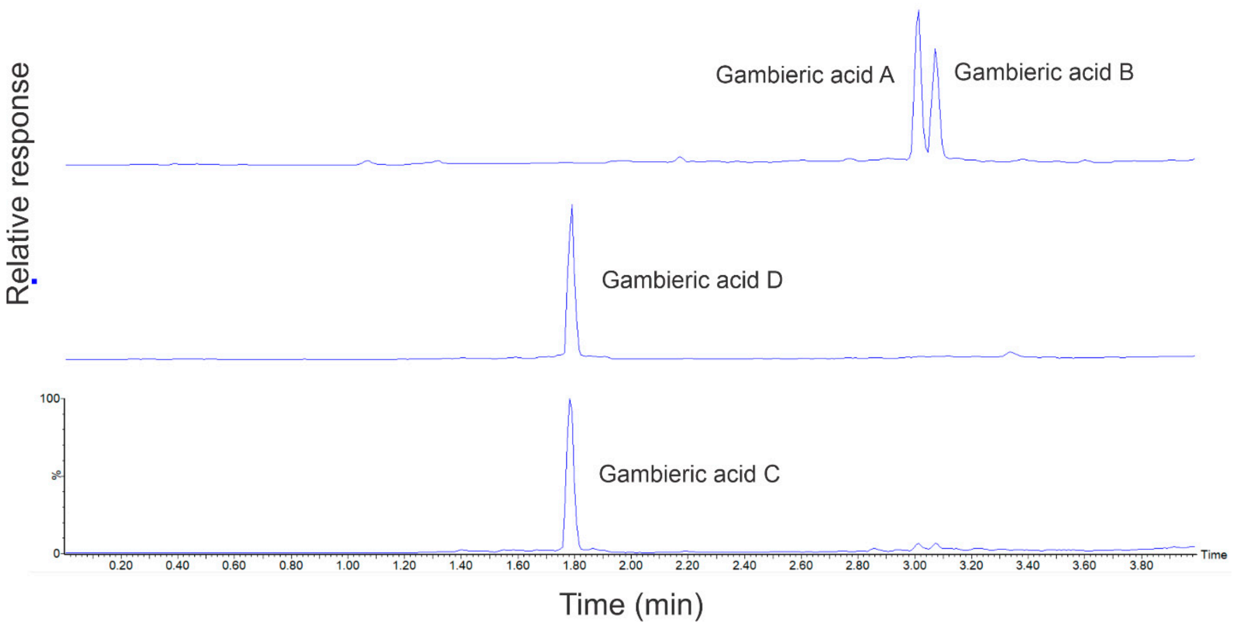

Figure S6. Example chromatogram showing the elution order of the gambieric acid metabolites.

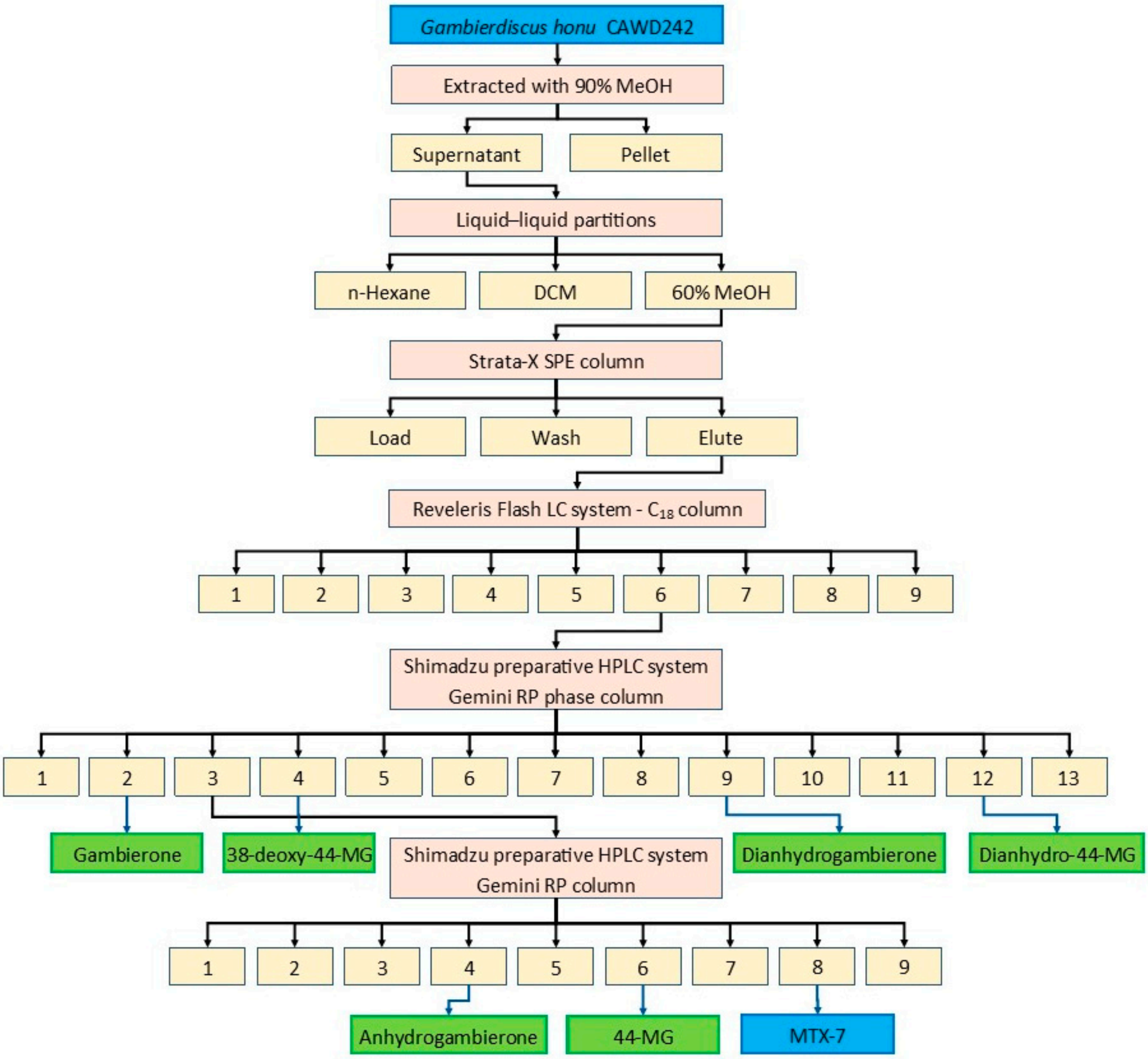

Figure S7. Purification scheme for the anhydro and dianhydro gambierone analogues

References

1. Yogi, K.; Sakugawa, S.; Oshiro, N.; Ikehara, T.; Sugiyama, K.; Yasumoto, T. Determination of toxins involved in ciguatera fish poisoning in the Pacific by LC/MS. *J. AOAC Int.* **2014**, *97*, 398-402, doi:10.5740/jaoacint.SGEYogi.
2. Yokozeki, T.; Hama, Y.; Fujita, K.; Igarashi, T.; Hirama, M.; Tsumuraya, T. Evaluation of relative potency of calibrated ciguatoxin congeners by near-infrared fluorescent receptor binding and neuroblastoma cell-based assays. *Toxicon* **2023**, *230*, 107161, doi:https://doi.org/10.1016/j.toxicon.2023.107161.
3. Food and Agriculture Organisation of the United Nations. *Report of the expert meeting on ciguatera poisoning. Rome, 19–23 November 2018*; 2020; p 156.
4. Vernoux, J.P.; Lewis, R.J. Isolation and characterisation of Caribbean ciguatoxins from the horse-eye jack (*Caranx latus*). *Toxicon* **1997**, *35*, 889-900.
5. Murata, M.; Naoki, H.; Iwashita, T.; Matsunaga, S.; Sasaki, M.; Yokoyama, A.; Yasumoto, T. Structure of maitotoxin. *Journal of the American Chemical Society* **1993**, *115*, 2060-2062, doi:10.1021/ja00058a075.
6. Caillaud, A.; Yasumoto, T.; Diogene, J. Detection and quantification of maitotoxin-like compounds using a neuroblastoma (Neuro-2a) cell based assay. Application to the screening of maitotoxin-like compounds in *Gambierdiscus* spp. *Toxicon* **2010**, *56*, 36-44, doi:10.1016/j.toxicon.2010.03.012 [doi].
7. Holmes, M.J.; Lewis, R.J. Purification and characterisation of large and small maitotoxins from cultured *Gambierdiscus toxicus*. *Natural Toxins* **1994**, *2*, 64-72.
8. Murray, J.S.; Finch, S.C.; Mudge, E.M.; Wilkins, A.L.; Puddick, J.; Harwood, D.T.; Rhodes, L.L.; van Ginkel, R.; Rise, F.; Prinsep, M.R. Structural characterization of maitotoxins produced by toxic *Gambierdiscus* species. *Marine drugs* **2022**, *20*, 453.
9. Murray, J.S.; Finch, S.C.; Puddick, J.; Rhodes, L.L.; Harwood, D.T.; van Ginkel, R.; Prinsep, M.R. Acute toxicity of gambierone and quantitative analysis of gambierones produced by cohabitating benthic dinoflagellates. *Toxins* **2021**, *13*, 333.
10. Stuart, J.; Smith, K.F.; Rhodes, L.; Murray, J.S.; Viallon, J.; Henry, K.; Darius, H.T.; Murray, S.A.; De Azevedo, C.D.; Argyle, P.; Chinain, M. Geographical distribution, molecular and toxin diversity of the dinoflagellate species *Gambierdiscus honu* in the Pacific region. *Harmful Algae* **2022**, *118*, 102308, doi:https://doi.org/10.1016/j.hal.2022.102308.
11. Fuwa, H.; Kainuma, N.; Satake, M.; Sasaki, M. Synthesis and biological evaluation of gambierol analogues. *Bioorganic Medical Chemistry Letters* **2003**, *13*, 2519-2522.
12. Nagai, H.; Murata, M.; Torigoe, K.; Satake, M.; Yasumoto, T. Gambieric acids, new potent antifungal substances with unprecedented polyether structures from a marine dinoflagellate *Gambierdiscus toxicus*. *The Journal of Organic Chemistry* **1992**, *57*, 5448-5453, doi:10.1021/jo00046a029.
